# Scoring information integration with statistical quality control enhanced cross-run analysis of data-independent acquisition proteomics data

**DOI:** 10.1038/s42004-025-01734-5

**Published:** 2025-11-20

**Authors:** Mingxuan Gao, Shubham Gupta, Wenxian Yang, Rongshan Yu, Hannes L. Röst

**Affiliations:** 1 Terrence Donnelly Centre for Cellular & Biomolecular Research, University of Toronto, Toronto, Canada; 2 Department of Molecular Genetics, University of Toronto, Toronto, Canada; 3 School of Informatics, Xiamen University, Xiamen, China; 4 National Institute for Data Science in Health and Medicine, Xiamen University, Xiamen, China; 5 Department of Genetics, Stanford University, Stanford, USA; 6 Aginome Scientific, Xiamen, China; 7 Department of Computer Science, University of Toronto, Toronto, Canada

**Keywords:** Mass spectrometry, Proteomics

## Abstract

The peptide-centric strategy is widely applied in data-independent acquisition (DIA) proteomics to analyze multiplexed MS2 spectra. However, current software tools often rely on single-run data for peptide peak identification, leading to inconsistent quantification across heterogeneous datasets. Match-between-runs (MBR) algorithms address this by aligning peaks or elution profiles post-analysis, but they are often ad hoc and lack statistical frameworks for controlling peak quality, causing false positives and reduced quantitative reproducibility. Here we present DreamDIAlignR, a cross-run peptide-centric tool that integrates peptide elution behavior across runs with a deep learning peak identifier and alignment algorithm for consistent peak picking and FDR-controlled scoring. DreamDIAlignR outperformed state-of-the-art MBR methods, identifying up to 21.2% more quantitatively changing proteins in a benchmark dataset and 36.6% more in a cancer dataset. Additionally, DreamDIAlignR establishes an improved methodology for performing MBR compatible with existing DIA analysis tools, thereby enhancing the overall quality of DIA analysis.

## Abstract (web summary)

Data-independent acquisition (DIA) proteomics often struggles with inconsistent peptide quantification across heterogeneous datasets due to reliance on single-run data. Here, the authors introduce DreamDIAlignR, a cross-run peptide-centric tool that leverages deep learning for consistent peak picking and FDR-controlled scoring to improve peak identification and alignment, enhancing protein and peptide detection accuracy and reproducibility in DIA analyses.

## Introduction

The data-independent acquisition (DIA)-based liquid chromatography coupled with tandem mass spectrometry (LC-MS/MS) strategy enables accurate and reproducible protein identification and quantification for large-scale molecular biology research^1,2^. It is one of the most widely utilized high-throughput proteome profiling methods, often used for its superior performance in cross-run quantitative cohort studies^3–7^. During the DIA data acquisition process, all peptide precursors in a relatively wide mass-to-charge (m/z) ratio window are selected for cofragmentation in an unbiased manner^8^, resulting in highly multiplexed MS2 spectra that are not suitable for direct analysis by peptide search engines for the identification of peptides^9–11^. To address this issue, peptide-centric analysis (PECA) methods^12,13^ were developed, where a spectral library containing predefined m/z values, retention time (RT), fragment ion intensities, and all the necessary information of the peptides of interest is used to query against raw DIA data to find evidence of the presence of each peptide in the library at a certain confidence level^14^. In PECA, only the chromatograms of the peptides and fragment ions in the library are extracted from the raw data and analyzed in a targeted manner, injecting strong priors into the data analysis and helping to alleviate the interference from cofragmented ions and improve identification sensitivity.

Since the concept of targeted data extraction was introduced, several software tools have been developed for PECA on DIA proteomics data^15^. OpenSWATH^16^ pioneered automated DIA data analysis by extracting chromatograms (XICs), scoring co-eluting peak groups, and employing semi-supervised learning with FDR control^14,17^, establishing a foundational framework for subsequent tools^18^. DIA-NN^19^ built upon this foundation by incorporating additional sub-scores and leveraging a neural network model to enhance peptide identification. Taking these advancements further, DreamDIA^20^ replaced traditional scores with a deep learning model, demonstrating superior capability in capturing complex chromatogram features^21^.

Although existing PECA tools excel in single-run analysis, achieving consistent analyte identification and quantification across heterogeneous sample cohorts remains challenging^22–25^. Most existing algorithms treat each run independently, without integrating information from other runs^16,19,20^. When iterating over each run in a sample set one at a time, PECA tools attempt to find viable chromatographic signals, referred to as “peak groups”, for peptides in the spectral library sequentially and score these peak groups separately. However, these peak group scores reflect only the elution behavior of peptides in individual runs. Consequently, the target-decoy binary discriminative model relies solely on single-run peak group scores, overlooking the relationships among peak groups of the same peptide across all runs. This limitation can lead to inconsistent peak identification for a single peptide across different runs, especially when a high-scoring peak group originates from another peptide in one run but not in the other. Treating each LC-MS/MS run independently substantially hinders the ability of a statistical model to analyze large-scale datasets and correct for experimental idiosyncrasies in these datasets^25^. While Group-DIA^26^ attempts to leverage correlations between chromatograms across multiple runs, its effectiveness is limited to homogeneous datasets with highly similar elution signals. The fragmentation of information across runs remains a critical, unresolved challenge in computational proteomics, and overcoming it becomes essential for ensuring robust, reproducible analyses and advancing the field’s capacity to handle large-scale datasets.

To enhance cross-run peptide identification and quantification reproducibility, several match-between-runs (MBR) algorithms have been developed^19,22,23,27^. These MBR algorithms compare and align signals among multiple runs after regular PECA, effectively correcting the RT locations and boundaries of falsely identified peak groups after statistical scoring. However, despite these advances, MBR algorithms still rely on the peak groups picked and the scores assigned by single-run analysis approaches. Their performance depends on high-scoring “reference” peaks observed in one or a few runs. However, the peak with the highest single-run score across all runs may not always be the correct one due to variations in interfering signals across different runs. Consequently, if the reference peak is chosen incorrectly, the identified peaks in all runs could be completely erroneous. Moreover, MBR is typically applied after statistical scoring, and thus undermines the guarantees and safeguards provided by FDR control, potentially leading to a substantial increase of the number of false positive peak groups. Currently, there are no statistical methods available capable of estimating the identification confidence of aligned peaks. In practice, MBR algorithms often require running with a heuristically increased, less strict FDR. While this approach reports more candidate peak groups, it unintentionally leads to decreased quantification performance. Consequently, MBR algorithms face a dilemma in balancing the trade-off between identification and quantification: They must either accept more low-quality but well-aligned peaks, compromising quantification accuracy, or reduce identification numbers to improve quantification.

Here, we introduce DreamDIAlignR, a cross-run peptide-centric DIA proteomics data analysis software tool. It integrates the DreamDIA^20^ deep learning peak scorer with the MBR algorithm DIAlignR^22^, enabling consistent cross-run peptide identification at the entire dataset level. Instead of processing each MS injection sequentially, it considers all-run performance collectively for each library peptide. By aligning raw chromatograms first, it calculates a multi-run score for each peak, which is a weighted combination of single-run quality scores that reflects the overall performance across all runs in the dataset. Considering both single-run peak quality and multi-run global performance, the peak identification confidence can be automatically estimated using a statistical model between target peptides and decoys, eliminating the need for manual ad hoc tuning of the FDR threshold. Meanwhile, it enables the discriminative model to learn the alignment relationship among peak groups across runs, thereby reducing inconsistent cross-run peak selection. In experiments conducted on both standard datasets and highly heterogeneous datasets, DreamDIAlignR substantially outperformed state-of-the-art software tools, achieving more consistent identification and enhanced quantification accuracy. Moreover, our results show that analyzing highly heterogeneous DIA data across multiple runs simultaneously yields superior cross-run protein quantification results compared to using single-run-only methods.

## Results

### Design of the DreamDIAlignR workflow

**Fig. 1:**
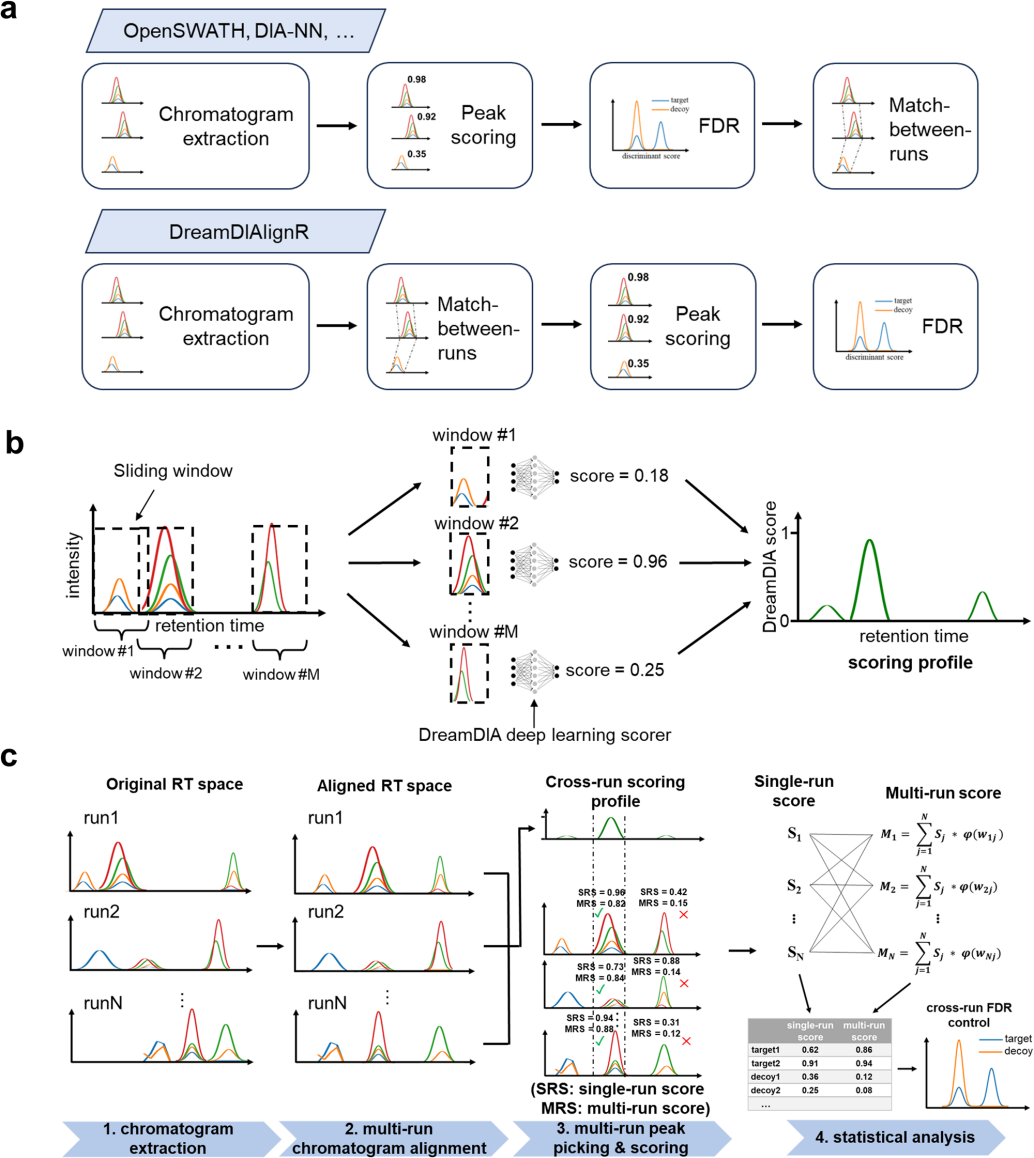
Schematic illustration of DreamDIAlignR. **a** Working principle of the DreamDIAlignR pipeline versus existing tools. Existing software, such as OpenSWATH and DIA-NN, perform match-between-runs (MBR) after statistical scoring, placing the matched peaks outside the scope of the original statistical error model. DreamDIAlignR instead aligns chromatographic signals before peak scoring, enabling rigorous, model-based confidence estimation (false discovery rate control) for the aligned peaks. **b** Working principle of the DreamDIA deep learning peak group scorer. First, a sliding window traverses the chromatogram time point by time point. Then, the signal within each window is fed into the pre-trained deep learning model to estimate its probability of being a real peptide signal (high score) or noise (low score). Finally, a continuous scoring profile is obtained for the corresponding chromatogram. **c** DreamDIAlignR cross-run peptide-centric analysis workflow. Instead of processing individual runs sequentially, DreamDIAlignR considers all runs together for each peptide. First, DreamDIAlignR extracts chromatograms from all runs and scores them using the DreamDIA deep learning peak scorer. Next, the MBR algorithm aligns the chromatograms and scoring profiles across runs. Then, the peaks are picked based on a majority voting of multiple runs represented by an averaged cross-run scoring profile instead of relying on a reference peak, and the multi-run scores are calculated by aggregating scores of corresponding peaks across runs. Finally, DreamDIAlignR considers both single-run scores and multi-run scores for statistical analysis.

DreamDIAlignR implements a general DIA data analysis workflow with match-between-runs (MBR) safeguarded by rigorous statistical control (Fig. 1a). Unlike existing tools, DreamDIAlignR performs MBR prior to FDR estimation, eliminating the need for ad hoc FDR tuning and ensuring that cross-run analysis adheres to a statistically principled quality control framework. The DreamDIAlignR workflow incorporates two recent innovations in targeted DIA analysis. First, it uses a deep learning-based method to generate a continuous quality scoring profile for peptide peak groups along the RT axis in each run. Second, a dynamic programming algorithm aligns chromatographic traces, ensuring one-to-one mapping of data points across runs. These strategies seamlessly integrate peptide identification information across all runs without discontinuity or hard cutoffs. The workflow comprises the following four main steps (Fig. 1c, see Methods for details).

#### Chromatogram extraction and scoring

DreamDIAlignR first extracts chromatograms of precursor and fragment ions across all runs for each library peptide. It then applies a pre-trained deep learning model to slide a scoring window along the RT, producing continuous scoring profiles that estimate the likelihood of each time point-and its surrounding context-representing a true peptide peak group (Fig. 1b). By default, DreamDIAlignR uses a Long Short-Term Memory (LSTM) model^28^ consisting of two LSTM layers^20^, which outputs a scalar score between 0 and 1 for each window. The model was trained on approximately one million chromatographic peaks collected from multiple instrument vendors to ensure robust and accurate peak scoring^20^.

#### Multi-run chromatogram alignment

To address RT misalignment caused by sample heterogeneity and experimental variations^29^, we then employ MBR algorithms to synchronize chromatograms and scoring profiles across runs, including both run-wide global alignment^27,30^ and peptide-wide dynamic alignment via DIAlignR^22,23^ to account for peptide-specific elution behavior.

#### Multi-run peak picking and peak scoring

By averaging aligned single-run scoring profiles, DreamDIAlignR generates a cross-run scoring profile that represents the collective elution behavior of each peptide across all runs. Candidate peak groups with the highest averaged scores are then picked, ensuring identification is based on majority consensus rather than relying solely on single-run data^16^. Simultaneously, we introduce a multi-run score for each peak group, which combines single-run scores weighted by global RT similarity, providing a balanced assessment of both individual and cross-run elution behavior to improve peak group identification.

#### Statistical analysis

The output of DreamDIAlignR is a discriminative model to distinguish between real peptides and artificially created decoys^14,17,19^. In contrast to routine methods that only concern peak groups’ single-run behavior, DreamDIAlignR extends existing statistical approaches^14,17^ to learn the peak group correspondence across runs by utilizing both single-run scores and multi-run scores, thereby enabling more consistent peak selection and more comprehensive confidence estimation with a cross-run horizon.

### Feasibility of cross-run signal alignment and integration

We first performed a feasibility test of our cross-run signal alignment and integration strategy using a pilot dataset, the *Streptococcus pyogenes* (*S. pyogenes*) dataset^27,31^, which included approximately 7000 manually annotated peak groups across 16 LC-MS/MS injections (Supplementary Fig. S20a).

**Fig. 2:**
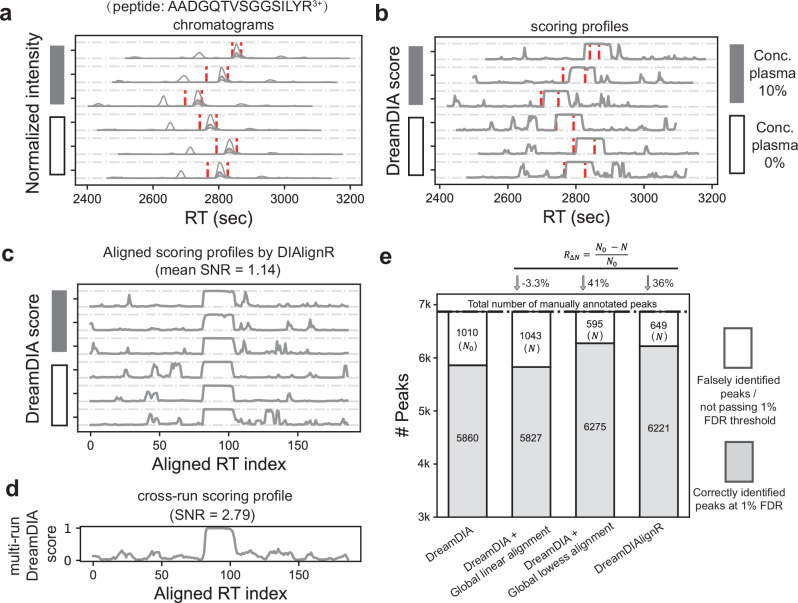
Chromatogram alignment and integration methods in the DreamDIAlignR workflow and their performance on a *Streptococcus pyogenes* (*S. pyogenes*) dataset. **a**–**d** Intermediate results of an example peptide “ADGQTVSGGSILYR^3+^” in the *S. pyogenes* dataset being processed by DreamDIAlignR. Results of 6 out of 16 runs are shown due to limited space. Gray boxes represent runs with 10% human plasma, while white boxes represent runs without human plasma. **a** Chromatograms of 500–1000 seconds wide are extracted in all runs. Red dashed lines denote manually annotated peak boundaries. **b** A continuous scoring profile is calculated for each run by DreamDIA deep learning peak group scorer. **c** Aligned scoring profiles of all runs by DIAlignR. **d** Averaged scoring profile of all runs. Signal-to-noise ratio (SNR) is calculated as the maximum score of manually annotated peak regions divided by the maximum score of the other regions. **e** Comparison of the number of correctly and incorrectly identified peak groups using different signal alignment methods. The incorrect identification number of DreamDIA without any alignment strategy is regarded as a baseline (*N*_0_), which is used to calculate the decreasing rates (*R*_*ΔN*_) when applying signal alignment algorithms.

As an example, the DreamDIA scoring profile for each run of the target peptide (Fig. 2b) is obtained by moving the DreamDIA peak group scorer along the RT axis of the corresponding chromatograms (Fig. 2a). Notably, the score apex regions show major concordance with the manually annotated peak group regions, indicating the peak identification accuracy of the DreamDIA scorer. In addition, although RT shifts among peak groups across multiple runs are clearly visible, the signals can be effectively synchronized when MBR algorithms like DIAlignR are applied (Fig. 2c). While all the MBR algorithms mitigate RT discrepancies across multiple runs, the chromatograms aligned by either global lowess method or DIAlignR demonstrate superior synchronization compared to the global linear method (Supplementary Fig. S1).

Moreover, although DreamDIA can correctly identify the locations of peak groups, there still remain some high-score noisy signals in the scoring profiles that fall outside the true peak group regions (Fig. 2b, c). To address this, we averaged the aligned scoring profiles from multiple runs. This process diluted and suppressed the random noise originating from a single experiment alone, resulting in a cross-run scoring profile with over 2-fold higher signal-to-noise ratio (SNR) compared to the single-run profiles (Fig. 2d). The averaged scoring profile will then be utilized for cross-run peak picking, which yields greater accuracy and consistency compared to sequential single-run peak picking methods.

We next evaluated the accuracy of peak group identification using manual annotations as ground truth, comparing different MBR approaches and DreamDIA without multi-run analysis (Fig. 2e). Compared to regular DreamDIA, global lowess alignment and DIAlignR reduced false identifications by 41% and 36%, respectively, while global linear alignment showed no improvement. This aligns with previous findings^22^, where global linear models failed to address run-to-run RT variation for this dataset. Non-linear lowess alignment also achieved lower residuals than linear alignment, demonstrating its robustness for datasets with significant RT shifts (Supplementary Fig. S2). DIAlignR, on the other hand, directly aligns chromatograms across runs for individual peptides. It provides the flexibility to adjust the alignment function for each peptide, resulting in comparable results to the global lowess alignment. It is noteworthy that different datasets exhibit varying cross-run RT discrepancy patterns, indicating that the performance of various MBR algorithms can vary across datasets. When choosing and benchmarking MBR algorithms, flexibility and robustness play a crucial role.

### Simultaneously improved identification and quantification performance

We benchmarked DreamDIAlignR’s peptide and protein identification and quantification performance using the LFQbench HYE110 dataset, which includes two replicated samples with known inter-species abundance ratios (human 1:1, yeast 10:1, and E. coli 1:10) for evaluating DIA tools’ ability to recover these ratios^32^ (Supplementary Fig. S20b).

**Fig. 3:**
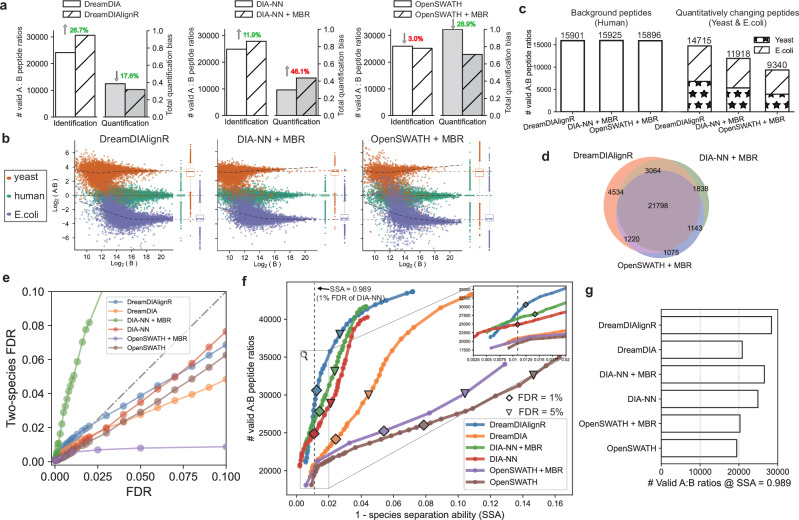
Identification and quantification performance benchmark on the LFQbench dataset. **a** Benchmark of match-between-runs (MBR) performance for DreamDIA, DIA-NN, and OpenSWATH. The number of valid peptide ratios and the total quantification bias before and after applying MBR at 1% precursor false discovery rate (FDR) are compared. Total quantification bias is computed as the geometric mean of three normalized metrics provided by the LFQbench software suite: 1 – species separation ability (SSA), median bias, and dispersion (see Methods). **b** Peptide-level LFQbench results for OpenSWATH + MBR, DIA-NN + MBR, and DreamDIAlignR at 1% precursor FDR. Colored dashed lines indicate log-transformed ground truth Sample A to Sample B ratios (human: 1:1; yeast: 10:1; E. coli: 1:10). Boxplot elements: center line, median; boxes, interquartile range; whiskers, 1.5 × interquartile range; points, outliers. **c** Number of valid peptide ratios identified for human, yeast, and E. coli. **d** Venn diagram showing the overlap of identified peptides across software tools. **e** FDR calibration curves of different software tools using the two-species method. An identical number of *Arabidopsis* peptides was spiked into a down-sampled LFQbench library as entrapment peptides to calculate the two-species FDR. The x-axis represents the FDR reported by each software tool, while the y-axis shows the actual two-species FDR, calculated as the total number of identifications divided by the number of identified entrapment peptides. **f** Total number of valid peptide ratios and corresponding 1 – SSA values across a range of FDR thresholds. SSA, as defined by the LFQbench package, is the area under the receiver operating characteristic (ROC) curve of a binary classifier separating species. Shown are the mean 1 – SSA values for human, yeast, and E. coli peptides. “♢” and “▿” indicate results at 1% and 5% precursor FDR, respectively. The vertical dashed line indicates performance at DIA-NN's 1% FDR quantification level, which serves as a benchmark for comparing the number of identifications across different software tools in (**g**). **g** Number of valid peptide ratios identified by each tool at DIA-NN's 1% FDR level (SSA = 0.989), as indicated by the dashed line in (**f**).

We first evaluated the performance of the MBR algorithms implemented in different software tools (Fig. 3a and Supplementary Fig. S3a). At 1% precursor FDR, DreamDIAlignR uniquely improved both identification and quantification, identifying 26.7% more peptides and reducing quantification bias by 17.6%. In contrast, DIA-NN’s MBR increased peptide identification by 11.9% but raised quantification bias by 46.1%. OpenSWATH’s MBR reduced quantification bias by 28.9% but at the expense of a slight 3.0% loss in peptide identification. Benchmarking at the protein level showed similar trends (Supplementary Fig. S3a). These findings indicate that both OpenSWATH and DIA-NN have to trade off quantitative accuracy with identification performance during MBR (with DIA-NN producing more, but quantitatively worse peptide identifications, while OpenSWATH produces less, but quantitatively better peptide identifications). However, this is not the case for DreamDIAlignR, which achieved better results in both.

Next, we benchmarked DreamDIAlignR’s overall performance with the state-of-the-art tools. DreamDIAlignR showed superior cross-run quantification accuracy compared to OpenSWATH and DIA-NN, with the A:B quantification ratios closer to the ground truth ratio line for each species and slightly higher dispersion than DIA-NN (Fig. 3b and Supplementary Fig. S3b). In many experimental settings, accurate identification and quantification of analytes that change in abundance are of particular interest since these could indicate proteins of interest for a drug target, biomarker or mechanistic study. We therefore focused our analysis next on the yeast and E. coli proteomes, which comprise the variable part of the A:B mixture experiment. Notably, all software tools yielded comparable numbers of human peptides and proteins, while DreamDIAlignR outperformed the other tools in identifying yeast and E. coli analytes (Fig. 3c and Supplementary Fig. S3c). It improved yeast and E. coli peptide precursor identifications by 23.5% and 57.5% over DIA-NN and OpenSWATH (Fig. 3c), and proteins by 21.2% and 47.3% (Supplementary Fig. S3c), respectively. The identified peptides and proteins that form valid A:B ratios show strong concordance with DIA-NN and OpenSWATH, with additional analytes uniquely identified by DreamDIAlignR, supporting the reliability of its results (Fig. 3d and Supplementary Fig. S3d). These additional peptides and proteins are predominantly from yeast and *E. coli*, indicating that DreamDIAlignR can reliably detect analytes across both high- and low-abundance samples to generate valid and accurate ratios (Supplementary Fig. S4). Manual inspection confirmed superior cross-run peak group identification consistency and quantification accuracy, particularly in low-abundance runs (Supplementary Fig. S5). These findings highlight DreamDIAlignR’s ability to detect more quantitatively changing proteins without compromising accuracy, offering significant advantages for multi-run analyses.

To estimate potential bias from overly optimistic FDR control by a single tool, we performed an entrapment analysis by spiking *Arabidopsis* peptides into the LFQbench library. Before MBR, all software tools exhibited well-calibrated FDRs, with the two-species FDR slightly lower than the reported FDR, indicating conservative estimation (Fig. 3e). However, DIA-NN’s MBR inflated the FDR by nearly threefold, reflecting a fundamental limitation of applying MBR after single-run-based FDR estimation. This approach bypasses the statistical calibration step for newly matched identifications, making it difficult to maintain reliable FDR control and potentially leading to inflated confidence in the results^33,34^. In contrast, OpenSWATH’s MBR produced overly conservative FDR estimates, likely due to the ad hoc removal of low-confidence identifications. DreamDIAlignR, however, maintained a well-calibrated FDR closely matching the two-species FDR, underscoring the robustness and accuracy of its FDR control strategy.

Next, we analyzed performance across a broad range of quality thresholds by evaluating the number of identified peptides versus the quantitative accuracy achieved on the benchmark dataset at different FDR cutoffs. This approach enables direct comparison, even in cases where the self-reported FDR is not well-calibrated, as observed with tools such as DIA-NN and OpenSWATH with MBR. The results showed that DreamDIAlignR substantially improved MBR performance and achieved the best overall results among all tools, with or without the MBR strategy (Fig. 3f and Supplementary Fig. S6). Across commonly used FDR thresholds ranging from 1% to 5%, DreamDIAlignR identified more analytes while maintaining superior quantification accuracy. To facilitate a more intuitive comparison, we added a vertical cutoff line at DIA-NN’s default 1% FDR level, enabling a fair comparison of the number of peptides identified by different tools at a consistent quantification accuracy. At a species separation ability (SSA) of 0.989, DreamDIAlignR identified 6.8% and 40.0% more peptides than DIA-NN with MBR and OpenSWATH with MBR, respectively (Fig. 3g). Results using alternative quantification metrics and cutoff values showed consistent trends (Supplementary Fig. S7). Furthermore, benchmarking different MBR algorithms available in DreamDIAlignR showed that the DIAlignR algorithm outperformed the other alignment methods (Supplementary Fig. S8). Notably, even when benchmarked against DIA-NN under its inflated FDR setting, DreamDIAlignR still achieved superior performance.

### The multi-run score ensures reliable cross-run peptide identification

Statistical error control is essential for ensuring reliable multi-run proteomics analysis^14^. Lim et al.^33^ highlighted this by designing an entrapment experiment, showing that MBR algorithms for DDA data caused an 8-fold increase in false identifications, including the erroneous identification of yeast peptides in human-only samples in their 2-Sample, 2-Proteome Challenge^34^. To mitigate erroneous alignment, DreamDIAlignR employs an exponential weight decay function governed by a penalty parameter *k*, which calculates multi-run scores by assigning higher weights to similar runs while penalizing contributions from distant ones. This approach prioritizes relevant scoring information, minimizing false identifications and avoiding inflated scores, particularly in highly heterogeneous datasets.

**Fig. 4:**
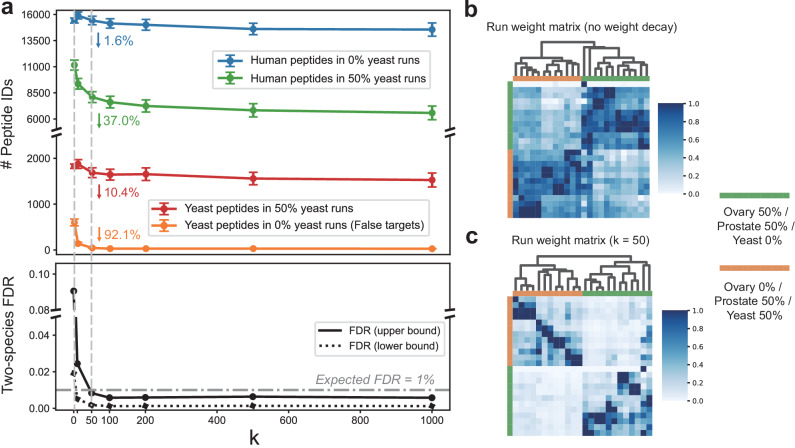
Optimization of weight decay parameters for multi-run score calculation on the Two-Sample, Two-Proteome (TSTP) dataset. **a** The number of peptide precursors identified and the corresponding two-species false discovery rate (FDR), calculated from mixed and human-only samples using various weight decay parameters, denoted as *k*. The parameter *k* controls the penalization of distant runs in the exponential weight decay function. A *k* value of 0 indicates that the weight decay approach has been deactivated. The number of yeast peptides identified in human-only runs serves as a measure of false targets. The two-species FDR was calculated using the “combined” method reported by Wen et al^35^ (See Methods). Error bars represent the standard deviation across 12 independent replicates (*n* = 12). **b** Run weight matrix without weight decay, showing hierarchical clustering based on similarity metrics. **c** Run weight matrix with weight decay (*k* = 50), showing hierarchical clustering based on similarity metrics.

Herein, we investigated the impact of the parameter *k* on the identification performance by using an entrapment experiment as well. A subset of 24 samples, consisting of 12 human-proteome-only runs and 12 human-yeast mixed runs from the Procan large-scale cancer study^25^, was selected for testing (Supplementary Fig. S20d). As *k* increased, the number of peptides identified declined, with a watershed *k* value of 50 (Fig. 4a). Compared to DreamDIAlignR without weight decay (*k* = 0), the number of entrapment (yeast) peptides falsely identified in the human-only runs decreased by 92.1% when *k* = 50. This reduction was significantly greater than the decreases observed in the yeast peptides identified in human-yeast mixed runs (10.4%) and human peptides in human-only runs (1.6%). We also monitored changes in FDR before and after applying the weight decay function. Two-species FDRs were calculated both with and without accounting for the differing likelihoods of human and yeast peptide identification, represented as the upper-bound and lower-bound FDRs^35^, respectively (See Methods). With a *k* value of 50, the upper-bound two-species FDR significantly decreased from 9.05% to 0.83%, while the lower-bound FDR also dropped from 1.91% to 0.18%. To further validate robustness, we conducted sensitivity tests across multiple datasets by tracking the total number of identifications at varying *k* values. A similar decreasing trend followed by a plateau was consistently observed, indicating the suppression of potential false positives arising from inflated multi-run scores (Supplementary Fig. S9). Based on this, we implemented an elbow-point method to automatically select an appropriate *k*, ensuring adaptability across datasets. Overall, these results demonstrate that the weight decay function substantially reduces false positives while preserving true identifications, enabling reliable cross-run FDR control.

In DreamDIAlignR, the final score assigned to a peak is a combination of its single-run score-reflecting the quality of its individual chromatographic signal-and a multi-run score, which captures the consistency of the peak across aligned runs. For the multi-run component, contributions from other runs are weighted according to the global RT similarity between the target run and each reference run. This strategy ensures that peaks from poorly matched runs do not disproportionately influence the multi-run score, even if they align with high-quality peaks. To validate this weighting scheme, we examined the run weight matrices before and after applying the weight decay function (Fig. 4b, c). As expected, highly similar runs (e.g., technical replicates) contributed more prominently to the multi-run score, while dissimilar runs (e.g., different sample types) were largely downweighted.

We also evaluated the impact of different global RT similarity metrics (see Methods) on cross-run performance. Among the four tested metrics, NC similarity consistently performed best: it effectively clustered similar runs, distinguished between different sample types-even those acquired on different instruments (Supplementary Fig. S10)-and resulted in more true identifications while maintaining fewer false identifications (Supplementary Fig. S11). Based on this evidence, NC similarity was selected as the default global RT similarity metric in DreamDIAlignR.

To assess whether false peptides in the library affect global RT similarity estimation, we conducted a spike-in experiment using varying amounts of entrapment *Arabidopsis* peptides^36^ in the LFQbench dataset. We found that DreamDIAlignR could still estimate global similarity accurately and robustly-even with library specificity reduced to 5%-provided a sufficient number of peptides were used for alignment (Supplementary Fig. S12).

In addition, the multi-run score, which indicates the overall quality of the aligned peaks across all runs, is a concept that current PECA software tools do not possess to the best of our knowledge. Therefore, we investigated whether the multi-run score could enhance the discriminative power of the statistical model to distinguish between target peptides and decoys. Results showed that the multi-run scores of yeast peptides in human-yeast mixed runs (true targets) were significantly higher than those in human-only runs (false targets) after applying weight decay (Supplementary Fig. S13a, b). Moreover, the multi-run score ranked second among all the sub-scores used by the statistical model, with a feature importance of 27.0% (Supplementary Fig. S13c). An ablation test also showed that discarding all multi-run scores before building the statistical model caused the number of identifications to revert to levels comparable to DreamDIA (Supplementary Fig. S13d). These results highlight the critical role of the multi-run score in helping the model accurately identify peptides in appropriate runs during entrapment testing.

### Better identification and quantification performance for highly heterogeneous datasets

One notable feature of DreamDIAlignR is its ability to analyze highly heterogeneous datasets through signal alignment and integration of multiple runs. Therefore, we evaluated its performance on the Procan^25^ cancer dataset, featuring mixed-species proteomes similar to LFQbench (Ovary 1:4, Prostate 1:1, Yeast 1.75:1) but with greater heterogeneity due to acquisition on different instruments over extended intervals (See Methods, Supplementary Fig. S20e).

**Fig. 5:**
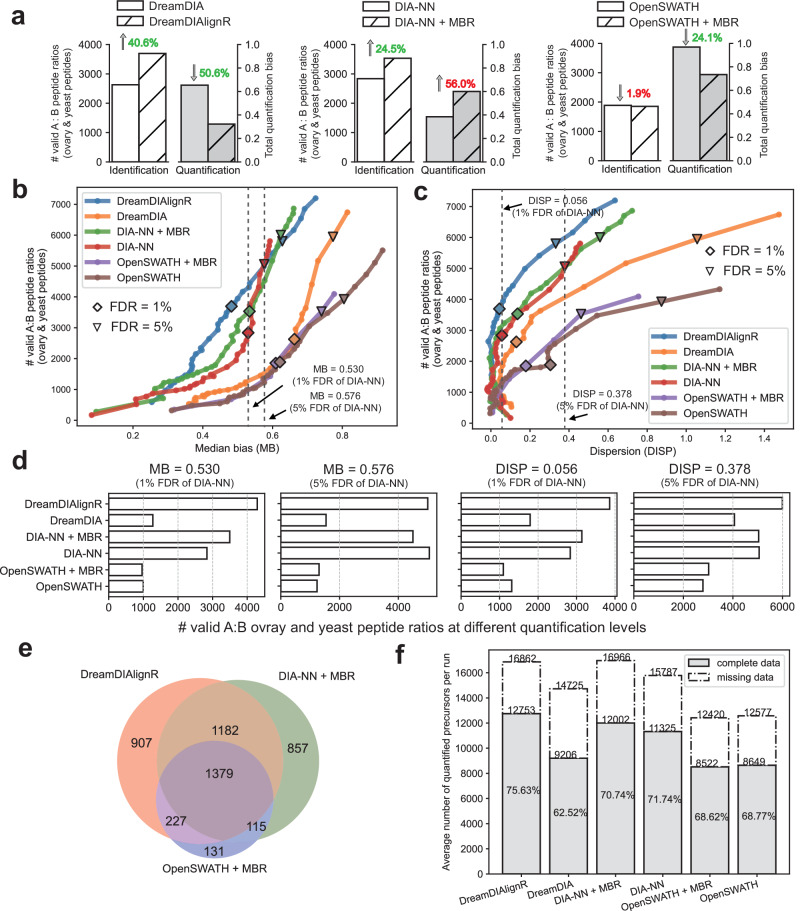
Identification and quantification performance benchmark on a highly heterogeneous dataset. **a** Performance comparison of match-between-runs (MBR) methods for DreamDIA, DIA-NN, and OpenSWATH at 1% precursor false discovery rate (FDR). Total quantification bias is computed as the geometric mean of two normalized quantification metrics: median bias (MB) and dispersion (DISP). Number of valid peptide ratios (Sample A vs. Sample B) for yeast and ovary peptides, plotted against the corresponding median bias (MB; **b**) and dispersion (DISP; **c**) across a range of FDR thresholds. A ratio is considered valid if the peptide is identified in at least 3 runs from both Sample A and Sample B. Data points represent the mean MB and DISP across yeast and ovary peptides. “♢” and “▿” indicate results at 1% and 5% precursor FDR, respectively. Vertical dashed lines denote quantification cut-offs at 1% and 5% FDR, based on DIA-NN, which were used to compare the number of identifications across different software tools in (**d**). **d** Number of valid yeast and ovary peptides identified by each tool at the benchmark quantification levels indicated in (**b**, **c**, **e**). Venn diagram showing overlap of identified peptides across software tools. **f** Benchmark of quantification matrix completeness. Percentages represent the proportion of validly quantified peptides out of all peptides identified. Solid bars denote the average number of quantified peptides per run; dashed bars show the total matrix dimensionality divided by the number of runs (36). Quantification matrices were filtered at 1% precursor FDR, and peptides identified in fewer than 3 runs were excluded.

Similar to the LFQbench analysis, we began by benchmarking the MBR algorithms across different software tools. At a 1% precursor FDR, DreamDIAlignR was the only MBR approach that consistently improved both identification and quantification performance. Compared to DreamDIA single-run analysis, DreamDIAlignR increased the number of identified peptides by 40.6% while reducing quantification bias by 50.6% (Fig. 5a). In contrast, OpenSWATH maintained a similar number of identifications but primarily reduced quantification bias by 24.1% after applying MBR. DIA-NN increased identifications by 24.5%, but at the cost of a 56.0% increase in quantification bias. Among all tools employing MBR, DreamDIAlignR achieved the highest identification count alongside the best quantification accuracy.

In the overall performance benchmark independent of FDR threshold selection, DreamDIAlignR significantly outperformed other tools in both identification and quantification (Fig. 5b, c). At equivalent quantification accuracy levels, DreamDIAlignR identified substantially more peptides than both OpenSWATH and DIA-NN-for example, 22.7% more at MB = 0.530 and 23.0% more at DISP = 0.056, compared to DIA-NN (Fig. 5d). Identifications were also consistent with those from other tools, confirming the reliability of the results (Fig. 5e). Among the three MBR algorithms implemented in DreamDIAlignR, DIAlignR showed the best performance for this dataset, yielding lower median bias than global alignment-based methods (Supplementary Fig. S14). Given the higher number of identified analytes, we further examined matrix completeness. DreamDIAlignR improved data completeness by 13.1% relative to DreamDIA single-run analysis, and by 4.9% and 7.0% compared to DIA-NN and OpenSWATH with MBR, respectively (Fig. 5f).

Moreover, to evaluate DreamDIAlignR on large-scale datasets, we analyzed the Procan494 dataset, consisting of 494 runs with technical replicates and varying species concentration ratios, enabling calibration curve generation (Supplementary Fig. S20f). Compared to DIA-NN, DreamDIAlignR demonstrated superior identification and quantification, identifying 21.2% more peptides and achieving a 2.7% higher median R^2^ at 1% FDR (Supplementary Fig. S15). This result highlights the robust performance of DreamDIAlignR in large-scale, high-throughput proteome profiling. We further assessed DreamDIAlignR using an LFQbench dataset acquired on an Orbitrap QE HF-X instrument with a different acquisition strategy-staggered all-ion fragmentation (AIF) (Supplementary Fig. S20c). In this setting, DreamDIAlignR again achieved the best overall identification and quantification performance (Supplementary Fig. S16a, b). When controlling for the number of identified peptides, DreamDIAlignR produced quantification ratios that were closer to the ground truth and exhibited lower dispersion than DIA-NN (Supplementary Fig. S16c, d). These results demonstrate the robustness of DreamDIAlignR across different instrument platforms and acquisition methods.

### Improved cross-run quantification provides more insightful data for biological analysis

Drawing meaningful biological conclusions in cohort proteomics studies hinges on the precise identification of differentially expressed signature proteins across samples. Achieving this requires robust and accurate cross-run peptide quantification, which enables the reliable detection of significant fold changes through statistical analysis amidst a large background of non-changing signals. Given that DreamDIAlignR has demonstrated superior cross-run quantification performance in benchmarking studies, we seek to evaluate its potential for providing deeper insights into biological changes in proteomic analyses.

**Fig. 6:**
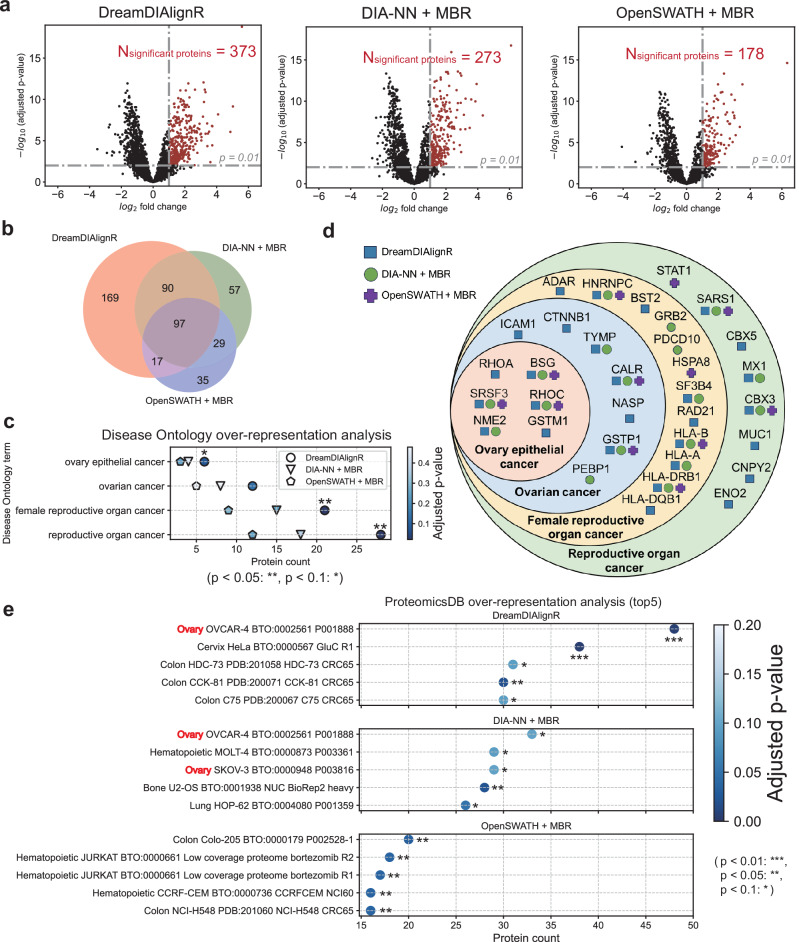
Comparison of differential expression analysis on the Two-Sample, Two-Proteome (TSTP) dataset conducted by three match-between-runs (MBR)-enabled software tools. **a** The numbers of differentially expressed proteins identified by DreamDIAlignR, DIA-NN with MBR and OpenSWATH with MBR in the 50% ovarian cancer tissue runs. The horizontal dashed lines indicate an adjusted p-value threshold of 0.01. The vertical lines represent a fold-change cut-off of 2. Proteins outside these thresholds are considered significantly up-regulated in the runs with 50% ovarian cancer tissue. **b** Consistency comparison of differentially expressed proteins identified by various software tools. **c** Over-representation analysis using Disease Ontology (DO) for the up-regulated proteins shown in **a**, highlighting DO terms related to ovarian cancer. **d** Ovarian cancer-related proteins were identified in the DO analysis using three different software tools. **e** Comparison of the top 5 over-represented gene sets in ProteomicsDB for the up-regulated proteins, identified using different software tools.

We conducted differential expression analysis on the 2-Sample, 2-Proteome dataset using limma^37^. Since only the Sample B runs contain ovarian cancer cells (Supplementary Fig. S20d), the up-regulated proteins in these runs can be considered ovarian cancer-related proteins. Our results demonstrated that DreamDIAlignR identified 36.6% and 109.6% more differentially expressed proteins at *p* < 0.01 using limma compared to DIA-NN with MBR and OpenSWATH with MBR, respectively (Fig. 6a). While a substantial overlap was observed in the proteins identified by the different software tools, DreamDIAlignR detected a greater number of unique proteins that are quantitatively changing (Fig. 6b). This suggests that DreamDIAlignR has a higher potential to uncover meaningful functional proteins or biomarkers in cohort-based proteomics studies.

To assess the biological relevance of the increased protein identifications by DreamDIAlignR, we performed over-representation analysis^38^ on the up-regulated proteins from the Sample B runs using two databases: Disease Ontology (DO)^39^ and ProteomicsDB^40^. In the DO analysis, the up-regulated proteins identified by DreamDIAlignR showed a stronger over-representation in ovarian cancer-related DO terms and yielded significantly lower p-values compared to the results from the other two software tools (Fig. 6c). Consistent with the overall overlap in differentially expressed proteins (Fig. 6b), DreamDIAlignR detected the majority of ovarian cancer-related proteins identified by the other two software tools, with its uniquely identified proteins also showing a strong association with ovarian cancer (Fig. 6d). Manual inspection of peaks identified by various software tools further showed that DreamDIAlignR delivered more complete and accurate cross-run quantification while effectively filtering out low-quality peaks (Supplementary Fig. S17, S18). This capability enhances differential expression analysis, providing higher statistical confidence compared to other tools.

Since Disease Ontology serves as a general semantic database primarily focused on linking genomic data with disease features and mechanisms^39^, we additionally utilized ProteomicsDB^40^-a database providing orthogonal disease information represented at the protein level-to perform over-representation analysis from a proteomics perspective. To ensure the completeness of the analysis results for different software tools, here we set the p-value cutoff for the over-representation analysis to 0.1 (while maintaining a 1% precursor FDR cutoff for all peptide identifications). Our results indicate that both DreamDIAlignR and DIA-NN with MBR rank OVCAR-4, a human ovarian cancer cell line, as the top gene set in the over-representation analysis (Fig. 6e). Notably, DreamDIAlignR identifies 45.5% more OVCAR-4 proteins than DIA-NN and achieves a significantly lower p-value (below 0.01). If the p-value cutoff is set to 0.05 or stricter, DreamDIAlignR remains the only tool capable of ranking the ovarian cancer cell line gene set first. Our analysis reveals that rather than merely improving the number of identifications, DreamDIAlignR strengthens DIA data analysis by enhancing the accuracy and coverage of identifying quantitatively changing analytes across samples. This capability supports the discovery of biologically meaningful signature proteins, providing valuable insights in real biomedical studies that involve highly heterogeneous data.

## Discussion

Consistently identifying and accurately quantifying peptides in biologically or technically heterogeneous samples remains not only the ultimate goal in DIA-based high-throughput proteomics research, but also a highly demanding task for all DIA data analysis tools^20^. Among the challenges encountered by DIA data analysis tools, a prominent one stems from the increased stochasticity of peptide elution signal selection across multiple samples, particularly when these samples have highly varied biological compositions or data acquisition conditions. Hence, the concept of harnessing multiple-run signal to alleviate interference and collectively identify chromatographic peaks is intuitive and has also been explored^26,41,42^. However, these existing tools were developed with the assumption of data homogeneity and only incorporated rudimentary signal alignment approaches, which were not suited for highly heterogeneous data. As a result, their use has been significantly limited in large-scale biomedical studies. To address this, current MBR algorithms^22,23,27^ prioritized fixing the retention time shift in heterogeneous data after a single-run analysis routine. However, they leave users with a dilemma: whether to accept aligned peaks of potentially lower quality or whether to reject potentially correct peaks to increase the stringency, due to the absence of a statistical framework for MBR. In essence, current “multi-run analysis tools” fall short of true multi-run capabilities, still confined by the conventional single-run analysis perspective.

In this study, we present an approach for performing MBR in DIA, which integrates data from all available LC-MS/MS runs and applies statistical error control after the match-between-runs, thereby ensuring that cross-run identification and quantification adhere to a rigorous statistical framework. This concept is in principle applicable to all DIA analysis tools, such as DIA-NN, OpenSWATH or Spectronaut, and addresses one of the last remaining challenges of DIA analysis: handling large-scale, heterogeneous study designs. Our findings demonstrate that DreamDIAlignR stands out as the only MBR method capable of simultaneously improving both identification and quantification performance, achieving a 36.6% increase in differentially expressed proteins in a realistic case-control dataset. Moreover, it is the only tool that reliably controls the FDR of aligned peaks by leveraging a rigorous statistical framework, rather than relying on ad hoc alignment strategies. While the Python-based implementation of DreamDIA introduces some computational overhead-primarily during single-run analysis-benchmark results show that DreamDIAlignR’s MBR runtime remains comparable to that of other tools (Supplementary Fig. S19). As LC-MS/MS technology and throughput continue to improve, we anticipate that DreamDIAlignR will not only assist proteomics researchers but also provide deeper insights into the analysis of large-scale heterogeneous omics data.

## Methods

### DreamDIAlignR workflow

DreamDIAlignR was developed based on DreamDIA^20^ and DIAlignR^22^ software libraries. The workflow of DreamDIAlignR includes four major steps described (See Fig. 1c) as follows.

#### chromatogram extraction and scoring

Before extracting chromatograms, DreamDIAlignR performs RT normalization using endogenous peptides sub-sampled from the spectral library. Initially, 4000 iRT peptides are randomly selected from the library by default. To ensure comprehensive iRT coverage and a representative sampling of the full library, the entire iRT range (e.g., −50 to 250) is divided into equal-width bins (layers), and peptides are randomly selected from each bin. Additionally, a 20% oversampling is applied to the first and last layers to improve model accuracy at the boundaries. Then, for each run, DreamDIAlignR identifies the RT locations with the highest scores for all the endogenous iRT peptides across the entire RT gradient using a pre-trained deep learning peak group scorer. This scorer is the built-in LSTM deep learning model in DreamDIA^20^, used without re-training or parameter fine-tuning. The RT location with the highest score is designated as the best RT. Peak groups located at these best RTs, with spectral cosine similarity scores above a certain threshold (0.95 by default), are considered validly identified iRT peptides. These best RTs are then used to fit a linear or non-linear model against their corresponding iRT values in the library. In DreamDIAlignR, the RT normalization step serves to both fit an RT versus iRT model to narrow down the peak group searching range^16,43,44^ and to obtain the global similarity among all runs. iRT peptides with fitting residuals below a specified threshold in each run are deemed confidently identified, or inliers. Global RT similarity between runs is then calculated based on the selected inlier peptides. DreamDIAlignR implements four similarity metrics: NC similarity, intensity similarity, XIC cosine similarity, and aligned XIC cosine similarity. The NC similarity^23^ between each run pair in the dataset is defined as:

$${w}_{ij}=2\frac{{N}_{{{\rm{common}}}}}{{N}_{{{{\rm{run}}}}_{i}}+{N}_{{{{\rm{run}}}}_{j}}}$$

where *w*_*ij*_ denotes the NC similarity, and *N*_common_ represents the number of common inlier peptide IDs in both run_*i*_ and run_*j*_. Intensity similarity is calculated as the cosine similarity of the total extracted MS2 ion intensities over the union of inliers. XIC cosine similarity is the mean cosine similarity of XICs for the union of inliers between two runs. Aligned XIC cosine similarity is similar to the XIC cosine similarity but uses aligned XICs for the comparison. In our benchmark, NC similarity performed best and is therefore set as the default. The similarity scores between all run pairs collectively form a global similarity matrix used for subsequent alignment steps.

Subsequently, DreamDIAlignR extracts the chromatograms for the top 6 fragment ions by default for all the target peptides and decoys in each run. With extracted chromatograms as input, the deep learning scorer slides along the RT ranging 200–1500 seconds, and scores the signals in each sliding window as a candidate peak group. To enhance the robustness and transferability of our approach, the scoring model also incorporates theoretical fragment ions based on the amino acid sequences of the peptides for peak group identification^20^. Consequently, both archived fragment ions from libraries and theoretical fragment ions have their respective chromatograms extracted in this process. DreamDIAlignR then calculates three additional single-run scores for each peak group except the DreamDIA deep learning score: spectral cosine similarity score, MS1 area score and MS2 area score. All these four scores create continuous traces along the RT axis, named as scoring profiles. The XICs and scoring profiles for each run are stored in SQLite database files for future use.

#### Multi-run chromatogram alignment

Due to sample heterogeneity and variations in experimental conditions across acquisitions, the XICs and scoring profiles of different runs often exhibit RT misalignment^29^. To make the chromatograms and scoring profiles of multiple runs comparable, we introduced MBR algorithms, including global RT alignment (run-wide) and DIAlignR alignment (peptide-wide). The global alignment strategy assumes a systematic RT variation between run pairs, where the elution order of peptides is preserved^27,30^. In DreamDIAlignR, the iRT peptides sub-sampled from the spectral library are initially selected as anchor peptides to build the global RT alignment models. These peptides are broadly distributed across the RT range and serve as a representative subset of the library. For each pair of runs, a global RT model is constructed using the best RTs of commonly and confidently identified anchor peptides. DreamDIAlignR provides both linear and lowess modeling options to capture RT correspondence between run pairs, enabling alignment of chromatograms for each peptide across all runs. However, this approach might smooth out peptide-specific RT shifts and lead to false identifications if the elution order of peptides differs across runs^30,45,46^. Therefore, we further introduced peptide-wide DIAlignR alignment that can be optionally applied on top of the global alignment constraint. DIAlignR aligns chromatograms for each peptide between two runs, but its time complexity, $O({N}_{{{\rm{peptides}}}}\times {N}_{{{\rm{runs}}}}^{2})$, can become prohibitive if all run pairs are aligned, especially in large sample cohorts. To address this, we constructed a minimum spanning tree based on the global similarity matrix, ensuring alignment only between run pairs connected by the tree’s edges^23^. This approach reduces the time complexity to *O*(*N*_peptides_ × *N*_runs_) and confines signal alignment to directly connected, highly similar runs, thereby minimizing false alignments and preventing information transfer between highly heterogeneous runs. After aligning chromatograms across multiple runs, the aligned RT vectors are interpolated and collapsed to produce a synchronized RT matrix for all runs. Users can flexibly choose between global RT alignment alone or the hybrid DIAlignR alignment. Benchmark results indicate that while the global RT alignment is more time-efficient, DIAlignR achieves higher accuracy.

#### Multi-run peak picking and peak scoring

With the RT of all runs aligned, the single-run scoring profiles are also synchronized. We then average the single-run scoring profiles across all runs to get the cross-run scoring profile. The top 10 candidate peak groups across all runs with the highest averaged deep learning scores are picked. This approach guarantees uniform peak selection and enhances comparability across the dataset.

For each candidate peak group, we compute multi-run scores to integrate evidence from all runs. Specifically, for each run, the multi-run score is calculated as a weighted linear combination of the corresponding single-run scores across runs. These single-run scores, including the deep learning score, spectral cosine similarity score, MS1 area score, and MS2 area score, are derived directly from the chromatographic signal, capturing peak shape, intensity, and coelution patterns. The resulting multi-run scores retain these characteristics and serve as robust indicators of peak quality across runs, helping to refine statistical confidence estimation. The weight of each single-run score in the linear combination is determined by the global RT similarity of the run it belongs to the target run. This strategy helps to avoid low-quality peak groups from being picked and accepted just because their multi-run scores have been over-boosted by high-quality peak groups in other runs. To further minimize false information transfer between distant runs, we introduced an exponential weight decay function, *ϕ*:

$$\phi (w)=\frac{{k}^{w}-1}{k-1}$$

where *w* represents the single-run score weight, and *k* is a parameter named as weight decay coefficient. This function significantly penalizes lower weight values while having minimal impact on higher weight values. As a result, single-run scores from highly similar runs predominantly contribute to the multi-run score. To improve robustness across diverse datasets, DreamDIAlignR implements an automated method to select an optimal *k*. It begins by downsampling the spectral library and analyzing the data using the reduced library, tracking the total number of identified peptides. As *k* increases, the number of peptide identifications initially drops sharply-reflecting the suppression of potential false positives caused by over-inflated multi-run scores-then enters a plateau phase where the number of IDs declines more gradually. The reduction curve is smoothed using a Gaussian filter, and the optimal *k* is determined by identifying the first elbow point.

Ultimately, we obtain four single-run scores, four multi-run scores, and several additional simple scores, such as delta RT score, peptide length, and charge for each peak group, preparing them for subsequent statistical analysis.

#### Statistical analysis

DreamDIAlignR constructs a semi-supervised learning model to distinguish between target peptides and decoys, utilizing either random forest or XGBoost algorithms. This approach mirrors the strategy employed by PyProphet^14^, with the key difference being the inclusion of multi-run scores in the analysis. The false discovery rate (FDR) is then estimated based on the distribution of discriminant scores between the target and decoy groups.

### Data preprocessing

All the DIA raw data files were converted to open file format mzML using MSConvert^47^ (version: 3.0.23080). Format conversion was conducted twice: once with the “Peak Picking” filter to obtain centroided files for DIA-NN, DreamDIA, and DreamDIAlignR, and once without the filter to produce profile files for OpenSWATH. For the Orbitrap LFQbench dataset, “Demultiplex” filter with “Overlap Only” option was applied to deconvolute the spectra prior to analysis. Software parameters used for all the experiments are shown in Supplementary Note 2.

### Feasibility test on the *S. pyogenes* dataset

The *S. pyogenes* dataset^16^ was acquired on a SCIEX TripleTOF 5600 instrument. It comprises a total of 16 runs, with 8 runs containing 10% human plasma as background and the remaining 8 runs lacking this component (Supplementary Fig. S20a). A spectral library built in the original paper^16,27,31^ was used. We manually checked the peak boundary annotation file to discard ambiguously and falsely annotated peaks (see Supplementary Note 1). Eventually, 6870 peaks were retained in total, including identification results from 434 peptides across 16 runs. Then we used the annotated peak locations to benchmark the peak identification performance of DreamDIAlignR. A correct identification is defined as an identified peak apex that falls into the range of the annotated peak boundaries.

### LFQbench test

The LFQbench HYE110 dataset^32^, acquired on the SCIEX TripleTOF 6600 instrument, was chosen for benchmarking (Supplementary Fig. S20b). To compare the quantification performance of different software tools across varying numbers of identified peptides, irrespective of hard FDR cutoffs, all tools were configured to output all results without applying any FDR filtering. Subsequently, the results were manually filtered using a range of FDR thresholds from 0.001 to 0.1. These filtered results were then input into the LFQbench software package to calculate quantification bias metrics, including 1 - species separation ability (SSA), median bias (MB), and dispersion (DISP). SSA is defined as the area under the receiver operating characteristic (ROC) curve of a binary classifier between two species. MB is defined as the distance between the median of quantified Sample A-to-Sample B ratios and the ground truth ratio lines. DISP is defined as the standard deviation of the log-transformed ratios for each species^32^. To illustrate overall quantification performance, we normalized the three quantification bias metrics using OpenSWATH as a baseline and defined total quantification bias as the geometric mean of these three normalized metrics at 1% FDR. To account for the impact of varying FDR thresholds on the identification results of OpenSWATH with MBR, we executed DIAlignR eight times after OpenSWATH analysis, each time specifying a different FDR threshold. To facilitate intuitive comparison across software tools, we introduced cut-off lines at specific quantification accuracy levels, enabling a direct assessment of the number of identified peptides at those thresholds. To avoid the impression of unfair optimization in favor of DreamDIAlignR, we used DIA-NN (without MBR)’s quantification levels at 1% and 5% precursor FDR as standardized benchmarks, given its well-calibrated FDR demonstrated in our evaluation. When exact values at these FDR thresholds were unavailable, the number of identifications was estimated by linear interpolation between neighboring FDR levels.

To assess whether cross-run analysis is affected by false peptides in the library, we performed an entrapment experiment using the LFQbench dataset. The original spectral library was first down-sampled to 8000 peptide precursors. A series of *Arabidopsis* peptides were then spiked into this library to generate libraries with varying specificity levels, ranging from 80% to 5%. These entrapment libraries were used to analyze the dataset with DreamDIAlignR and assess the impact on global RT alignment. Additionally, an entrapment library with 50% specificity was used to evaluate the FDR calibration of different software tools. Before analysis, *Arabidopsis* peptides sharing three or more fragment ion m/z values with true positive peptides (human, yeast, and *E. coli*) were removed to avoid ambiguous assignments. The two-species FDR was calculated as the number of identified *Arabidopsis* peptides divided by the total number of peptide identifications. All identified peptide precursors were included in the calculation without additional filtering.

To evaluate the robustness of DreamDIAlignR across different instrument vendors and acquisition methods, we further conducted a similar benchmarking experiment using an Orbitrap AIF LFQbench dataset (Supplementary Fig. S20c). Given the larger number of replicates in this dataset (nine per sample), peptides were included in the quantification ratio analysis only if identified in at least three runs from both Sample A and Sample B.

### Two-sample, two-proteome test

To create a two-sample, two-proteome entrapment experiment dataset, we selected 24 DIA runs from a previously published dataset, which had been used to evaluate analysis reproducibility of large-scale DIA proteomics studies^25^ (referred to as the TSTP dataset). The selected runs were technical replicates of two samples acquired on three different SCIEX TripleTOF 6600 mass spectrometers (#M2, #M4, and #M6) on two different days (Days 14 and 103). The 12 runs, consisting of 50% prostate cancer tissue and 50% yeast cell lysates, can be regarded as human-yeast mixed samples. Conversely, the remaining 12 runs with 50% ovarian cancer tissue and 50% prostate cancer tissue can be regarded as human-only samples (Supplementary Fig. S20d). The human spectral library and the yeast spectral library were combined to get the mixed library for analysis. Peptide precursors in the library were filtered to have the top 6 intense fragment ions. All the software tools were configured to output identification results at 1% precursor FDR. Peptide precursors detected in less than one-third of the runs or with intensities below 100 were excluded from the benchmark analysis. The two-species FDR was calculated using the “combined” method as described by Wen et al.^35^, with the following formulas:

$${{{\rm{FDR}}}}_{{{\rm{lowerbound}}}}=\frac{{N}_{{{\rm{yeast}}}}}{{N}_{{{\rm{yeast}}}}+{N}_{{{\rm{human}}}}}$$

$${{{\rm{FDR}}}}_{{{\rm{upperbound}}}}=\frac{{N}_{{{\rm{yeast}}}}(1+1/r)}{{N}_{{{\rm{yeast}}}}+{N}_{{{\rm{human}}}}}$$

Here, *N*_yeast_ and *N*_human_ deonte the numbers of human and yeast peptides identified in all the runs without the yeast component. The parameter *r* represents the effective ratio of yeast peptides in the mixed spectral library, which is 0.267 in this experiment. The upper-bound FDR provides a more conservative estimation, though it risks overestimating the actual FDR. Conversely, the lower-bound FDR may overlook the differences in the likelihood of identifying human versus yeast peptides^35^. To provide a more comprehensive assessment, both upper-bound and lower-bound FDRs were presented.

### Heterogeneous dataset tests

To comprehensively benchmark the identification and quantification performance of DreamDIAlignR on a highly heterogeneous dataset, we selected an additional 36 runs from the Procan cancer dataset^25^ (referred to as Procan36 dataset). This dataset also includes replicates of two distinct samples. Specifically, 18 runs contain 6.25% ovarian cancer tissue, 50% prostate cancer tissue, and 43.75% yeast cell lysates, while the other 18 runs contain 25%, 50% and 25% for the three portions, respectively (Supplementary Fig. S20e). Thus, the two sample sets allow us to establish ground truth species ratios (Ovary, 1:4; Prostate, 1:1; Yeast, 1.75:1) similar to those in the LFQbench dataset. The Procan36 dataset comprises DIA runs acquired on three different SCIEX TripleTOF 6600 mass spectrometers (#M1, #M3 and #M5) across two different days (Day14 and Day105). The diversity of cancer tissue introduces biological heterogeneity, while variations in experimental conditions result in technical heterogeneity. These factors collectively contribute to greater overall heterogeneity among runs compared to the LFQbench dataset. We computed quantification bias metrics, including median bias and dispersion, following the guidelines provided by the LFQbench^32^ software package. The total quantification bias was calculated in a manner similar to the LFQbench test, but without including species separation ability to avoid introducing bias from different metric calculation methods for the benchmark. Additionally, we defined ovary-specific peptides as those identified by DIA-NN (at a 1% FDR) in at least 8 out of 12 Ovary 50% runs but in at most 1 out of 12 Ovary 0% runs in the TSTP dataset. For data matrix completeness benchmarking, We filtered the quantification matrices yielded by all the software tools to include only analytes that had been identified in at least three runs and defined data completeness as the number of validly quantified entries divided by the total number of entries.

Furthermore, we selected a dataset of 494 runs from the Procan dataset to evaluate performance on a large-scale data (referred to as Procan494 dataset). This dataset includes replicates of four distinct samples, each with varying organism/species proteome ratios (Supplementary Fig. S20f). The data were acquired using 6 different SCIEX TripleTOF 6600 mass spectrometers (#M1, #M2, #M3, #M4, #M5, and #M6) across 7 different days (Day7, Day14, Day21, Day28, Day56, Day84, and Day107). To assess quantification performance, we calculated the median of the *R*^2^ values from the calibration curves for both ovarian peptides and yeast peptides. For identification performance, we defined a valid peptide as one identified in at least ten runs within each sample and then calculated the total number of valid peptides identified.

### Differential expression analysis and over-representation analysis

Differential expression analysis was performed on the TSTP dataset with a widely-accepted workflow^48^ as follows. Protein quantification matrices were generated by summing the peak areas of the top three most intense peptide precursors for each protein across DreamDIAlignR, DIA-NN with MBR, and OpenSWATH with MBR, all at 1% precursor FDR. Human peptides with intensities lower than 100, as well as all yeast peptides, were excluded. Additionally, peptide precursors identified in fewer than one-third of the runs were discarded. The remaining quantification matrices were imputed using k-nearest neighbors (KNN) algorithm with three neighbors. Following imputation, the matrices were quantile-normalized, log-transformed, and then analyzed with limma^37^ for protein signature identification and statistical analysis.

Lastly, we utilized the ClusterProfiler^38,49,50^ R package to perform over-representation analysis for the differentially expressed proteins using two different databases: Disease Ontology (DO)^39^ and ProteomicsDB^40,51^. For the DO analysis, we focused on ovarian cancer-related DO terms to facilitate comparison across different software tools. In the ProteomicsDB enrichment analysis, we selected the top five enriched gene sets with adjusted *p*-values below 0.1 as benchmarks for evaluation.

### Ethics

This study does not involve human participants, animal subjects, or data collection requiring ethical approval. Inclusion and diversity considerations are not applicable.

## Supplementary information

Transparent Peer Review file

Supplementary Information

## Data Availability

All the datasets used in this work are published and publicly available. The *S. pyogenes* dataset was deposited to PeptideAtlas with accession code PASS00788. The LFQbench dataset, Procan dataset, and the Orbitrap LFQbench dataset were deposited to ProteomeXchange Consortium^52,53^ with dataset identifiers PXD002952, PXD015912, and PXD028735, respectively. The code and data used to generate the figures have been uploaded to Zenodo (10.5281/zenodo.16284655).

## Code availability

DreamDIAlignR is implemented in Python and integrated in the DreamDIA^20^ software package (version 3.2.0). DreamDIAlignR was benchmarked with OpenSWATH^16^ (v2.7), DIAlignR^22,23^ (v2.6.0), DreamDIA^20^ (v2.0.3) and DIA-NN^19^ (v1.8). Software parameters used for all the experiments are shown in Supplementary Note 2. DreamDIAlignR is fully open-source under the Apache-2.0 license and available at https://github.com/xmuyulab/DreamDIA.
