# Supplementary material for: Scoring information integration with statistical quality control enhanced cross-run analysis of data-independent acquisition proteomics data
Source: Commun Chem. 2025 Nov 20;8:364. doi: 10.1038/s42004-025-01734-5 (PMC12635196; doi:10.1038/s42004-025-01734-5)
Supplement: Supplementary file 2 — Supplementary Information [file 42004_2025_1734_MOESM2_ESM.pdf]

Supplementary Information

Scoring Information Integration with Statistical Quality Control Enhanced Cross-Run Analysis of Data-Independent Acquisition Proteomics Data

Mingxuan Gao[1,2,3,4], Shubham Gupta[1,2,5], Wenxian Yang[6], Rongshan Yu[3,4,6✉], and Hannes L. Röst[1,2,7✉]

[1] Terrence Donnelly Centre for Cellular & Biomolecular Research, University of Toronto, Toronto, Canada

[2] Department of Molecular Genetics, University of Toronto, Toronto, Canada

[3] School of Informatics, Xiamen University, Xiamen, China

[4] National Institute for Data Science in Health and Medicine, Xiamen University, Xiamen, China

[5] Department of Genetics, Stanford University, Stanford, U.S.A

[6] Aginome Scientific, Xiamen, China

[7] Department of Computer Science, University of Toronto, Canada

Supplementary Notes

Supplementary Note 1: Excluded Peptide Annotations in S.pyogenes Dataset

We double-checked the manually annotated peak boundaries of the 400 peptides in S.pyogenes dataset by previously published work. We found that some of the annotated peak boundaries were confusing or wrong. Thus, we chose to exclude the following peptide annotations in our benchmark experiments.

ATGEVMAIGR2
TPLFNLLK2
LLIIDHR2
VVSM(UniMod:35)PSQNIFDEQSAEYKESILPAAVTK3
LVLDDLTFSIPK2
AAQALANTIIDHGPEAVKK3
IDKPGANPER2
INETKDFLVTK2
EQGYDVIGVFMK2
RVLESGQDYTM(UniMod:35)DPMSNSER3

Supplementary Note 2: Software Parameters

	S.pyogenes dataset	LFQbench dataset	TSTP dataset	Procan36 dataset / Procan494 dataset	OrbitrapLFQ dataset
**OpenSWATH**	/	-min_upper_edge_dist 1 -extra_rt_extraction_window 100 – mz_extraction_window 30 -Scoring:TransitionGroupPicker:background_subtraction exact	-min_upper_edge_dist 1 -extra_rt_extraction_window 100 – mz_extraction_window 30 -Scoring:TransitionGroupPicker:background_subtraction exact – Library:retentionTimeInterpretation seconds	-min_upper_edge_dist 1 -extra_rt_extraction_window 100 – mz_extraction_window 30 -Scoring:TransitionGroupPicker:background_subtraction exact – Library:retentionTime Interpretation seconds	-Scoring:TransitionGroupPicker:background_subtraction exact
**DIAlignR (OpenSWATH's MBR)**	/	(If the wanted FDR is x)<br>Params <- paramsDIAlignR()<br>params[["alignedFDR1"]] <- x<br>params[["alignedFDR2"]] <- x<br>params[["maxFdrQuery"]] <- x<br>params[["baseSubtraction"]] <- TRUE<br>params$smoothPeakArea <- TRUE<br>(Then run `mstAlignRuns` function)	(If the wanted FDR is x)<br>Params <- paramsDIAlignR()<br>params[["alignedFDR1"]] <- x<br>params[["alignedFDR2"]] <- x<br>params[["maxFdrQuery"]] <- x<br>params[["baseSubtraction"]] <- TRUE<br>params$smoothPeakArea <- TRUE<br>(Then run `mstAlignRuns` function)	(If the wanted FDR is x)<br>Params <- paramsDIAlignR()<br>params[["alignedFDR1"]] <- x<br>params[["alignedFDR2"]] <- x<br>params[["maxFdrQuery"]] <- x<br>params[["baseSubtraction"]] <- TRUE<br>params$smoothPeakArea <- TRUE<br>(Then run `mstAlignRuns` function)	(If the wanted FDR is x)<br>Params <- paramsDIAlignR()<br>params[["alignedFDR1"]] <- x<br>params[["alignedFDR2"]] <- x<br>params[["maxFdrQuery"]] <- x<br>params[["baseSubtraction"]] <- TRUE<br>params$smoothPeakArea <- TRUE<br>(Then run `mstAlignRuns` function)
**DreamDIA /DreamDIAlignR**	--mz_tol_ms1 0.05 --mz_tol_ms2 0.05 --n_cycles 200	default	--irt_mode rt	--irt_mode rt	--rt_tol 2.897
**DIA-NN + MBR**	/	Precursor FDR 100%<br>MBR: checked<br>Cross-run normalization: RT-dependent	MBR: checked<br>Cross-run normalization: RT-dependent	Precursor FDR 100%<br>MBR: checked<br>Cross-run normalization: RT-dependent	Precursor FDR 100%<br>MBR: checked<br>Cross-run normalization: RT-dependent
**DIA-NN**	/	Precursor FDR 100%<br>MBR: unchecked<br>Cross-run normalization: Off	MBR: unchecked<br>Cross-run normalization: Off	Precursor FDR 100%<br>MBR: unchecked<br>Cross-run normalization: Off	Precursor FDR 100%<br>MBR: unchecked<br>Cross-run normalization: Off

Supplementary Figures

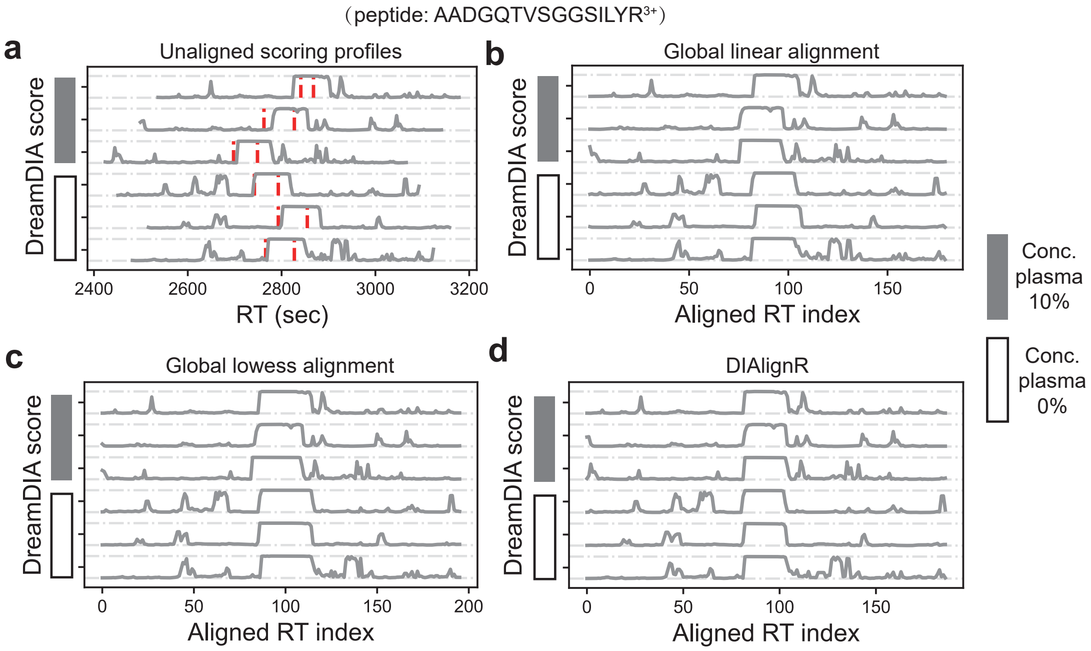

Figure S1. Scoring profile sychronization performance of an example peptide AADGQTVSGGSILYR^{3+} in Streptococcus Pyogenes dataset using different signal alignment methods. a. Unaligned scoring profiles. **b.** Aligned scoring profiles using linear global alignment. **c.** Aligned scoring profiles using lowess global alignment. **d.** Aligned scoring profiles using DIAlignR. Gray boxes represent runs with 10% human plasma, while white boxes represent runs without human plasma. Red dashed lines denote manually annotated peak boundaries. Only results of 6 out of 16 runs are shown due to the limit of figure space.

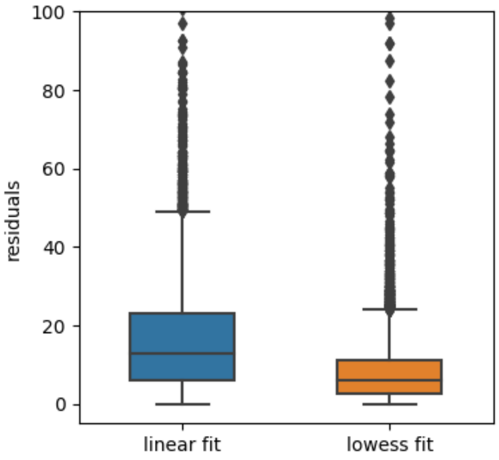

Figure S2. Residuals of linear or lowess global alignment methods on S.pyogenes dataset. A linear or lowess fit was built between the RT values of anchor peptides from each run pair in the global minimum spaning tree. Residuals of all the anchor peptides in all the fit models were plotted (linear fit: n = 8988; lowess fit: n = 6580). Residuals higher than 100 were discarded. Boxplot elements: center line, median; boxes, interquartile range; whiskers, 1.5x interquartile range; points, outliers.

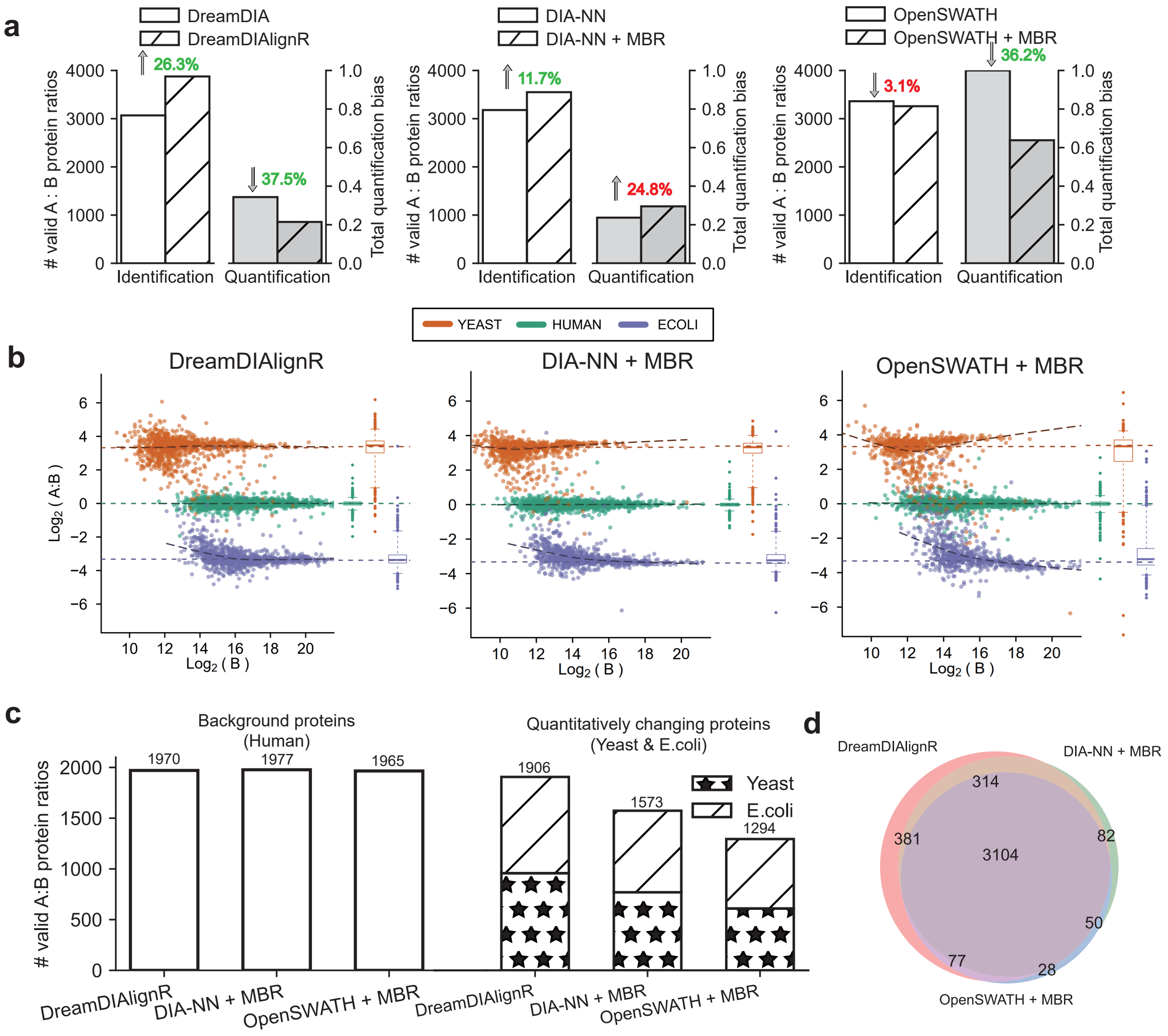

Figure S3. Identification and quantification performance benchmark on the LFQbench dataset (protein level). a Benchmark of match-between-runs (MBR) performance for DreamDIA, DIA-NN, and OpenSWATH. The number of valid protein ratios and the total quantification bias before and after applying MBR at 1% precursor FDR are compared. Total quantification bias is computed as the geometric mean of three normalized metrics provided by the LFQbench software suite: 1 – species separation ability, median bias, and dispersion (see Methods). **b** Protein-level LFQbench results for OpenSWATH + MBR, DIA-NN + MBR, and DreamDIAlignR at 1% precursor FDR. Colored dashed lines indicate log-transformed ground truth Sample A to Sample B ratios (Human: 1:1; Yeast: 10:1; *E. coli*: 1:10). Boxplot elements: center line, median; boxes, interquartile range; whiskers, 1.5× interquartile range; points, outliers. **c** Number of valid protein ratios identified for Human, Yeast, and *E. coli*. **d** Venn diagram showing the overlap of identified proteins across software tools.

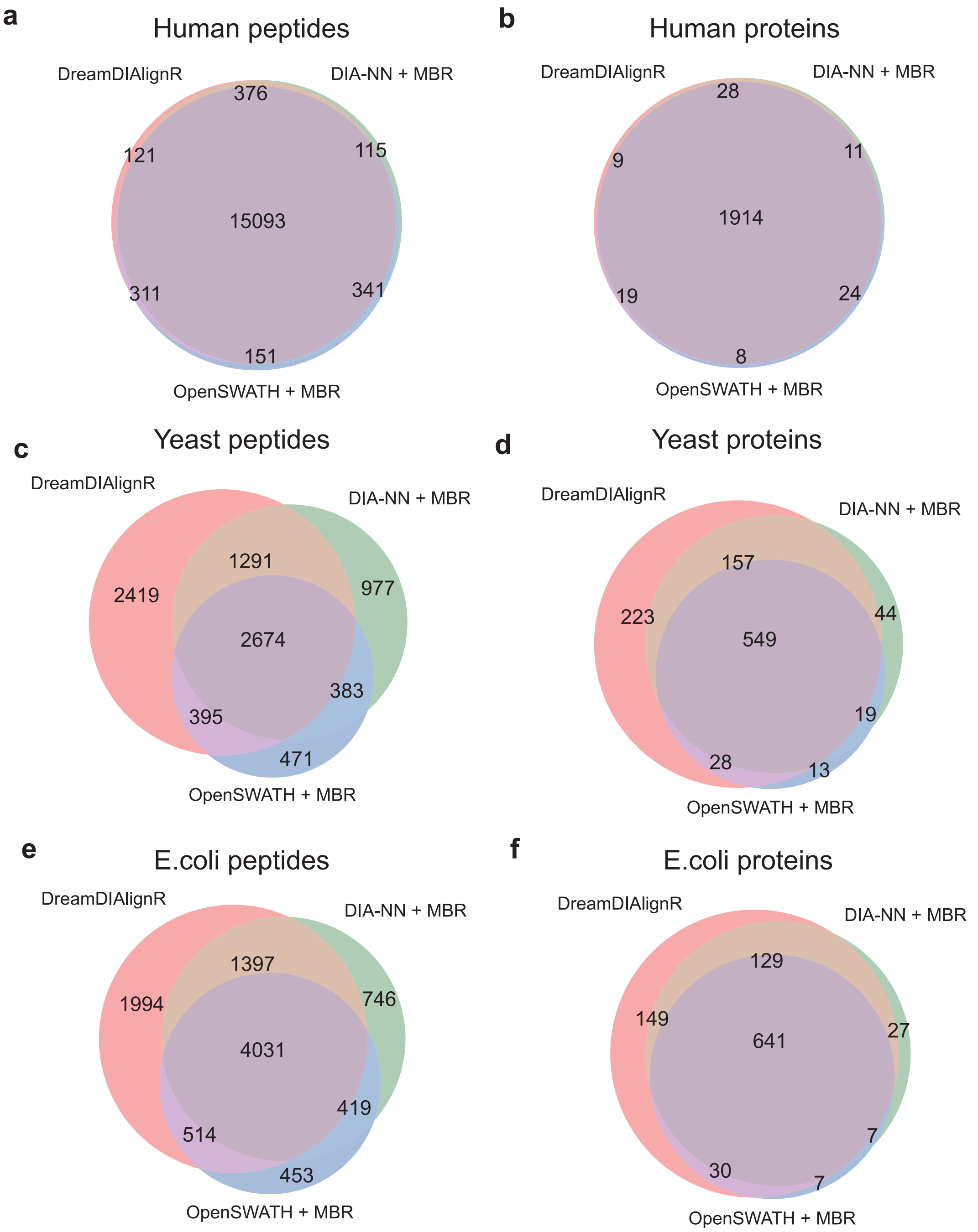

Figure S4. Venn diagrams showing the overlap of identified peptides and proteins for different species across software tools. a. Human peptides, **b.** Human proteins, **c.** Yeast peptides, **d.** Yeast proteins, **e.** *E. coli* peptides, **f.** *E. coli* proteins.

a

Peptide: VAPTTGVVAK^{2+} (Yeast)
Ground truth quantity ratio A:B = 10:1

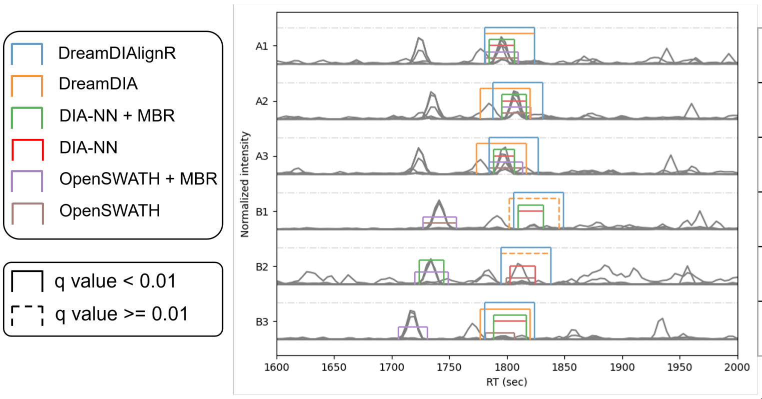

Quantification table

DreamDIAlignR	DreamDIA	DIA-NN + MBR	DIA-NN	OpenSWATH + MBR	OpenSWATH
79548	79548	19026	19026	25057	25057
87003	84113	20625	20625	28911	28911
76412	70047	16747	18221	24010	24010
7339	NaN (6125)	1945	1945	15769	15769
8517	NaN (8517)	12412	1598	19034	3658
6684	NaN (6034)	1447	1522	14846	963

b

Peptide: AYLHYIPK^{2+} (Yeast)
Ground truth quantity ratio A:B = 10:1

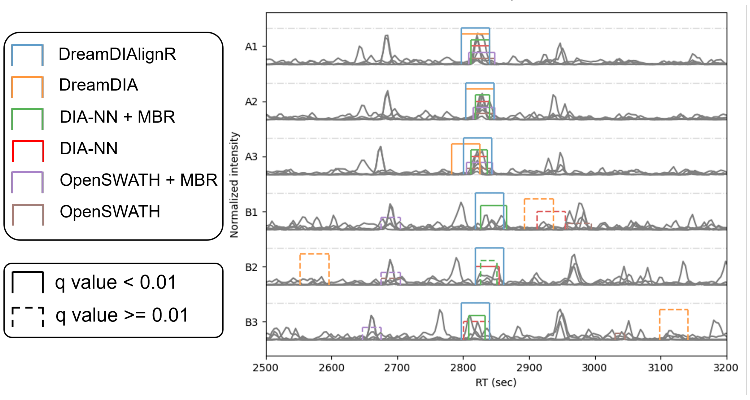

Quantification table

DreamDIAlignR	DreamDIA	DIA-NN + MBR	DIA-NN	OpenSWATH + MBR	OpenSWATH
67488	67488	16897	7612	20925	20925
61356	61356	13835	6779	11764	11764
56379	34710	14448	5732	17979	17979
6302	NaN (2402)	3530	NaN (690)	NaN (42553)	NaN (9586)
10447	NaN (3352)	NaN (2209)	1831	NaN (32209)	NaN (32209)
10135	NaN (1405)	3521	NaN (1794)	NaN (37286)	NaN (0)

c

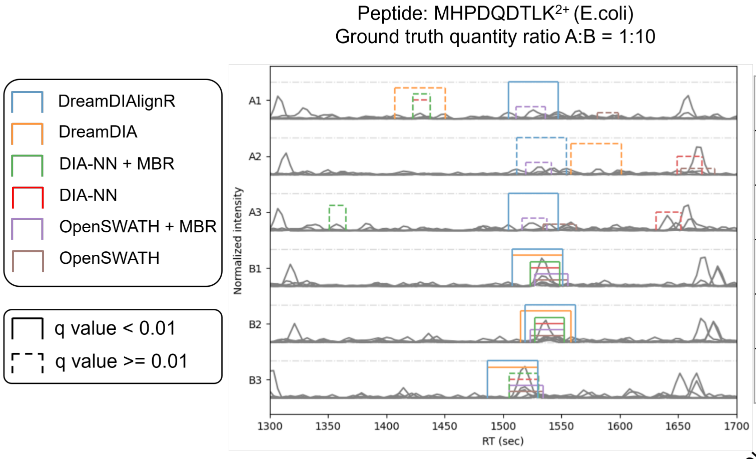

Quantification table

DreamDIAlignR	DreamDIA	DIA-NN + MBR	DIA-NN	OpenSWATH + MBR	OpenSWATH
828	NaN (1204)	NaN (228)	NaN (228)	NaN (587)	NaN (193)
NaN (2388)	NaN (416)	NaN	NaN (327)	NaN (416)	NaN (1592)
629	NaN (1272)	NaN (191)	NaN (363)	NaN (386)	NaN (1241)
11567	11567	3386	3386	1593	1593
7613	8810	3491	3491	3987	3987
8754	5849	3735	NaN (3667)	3173	3173

d

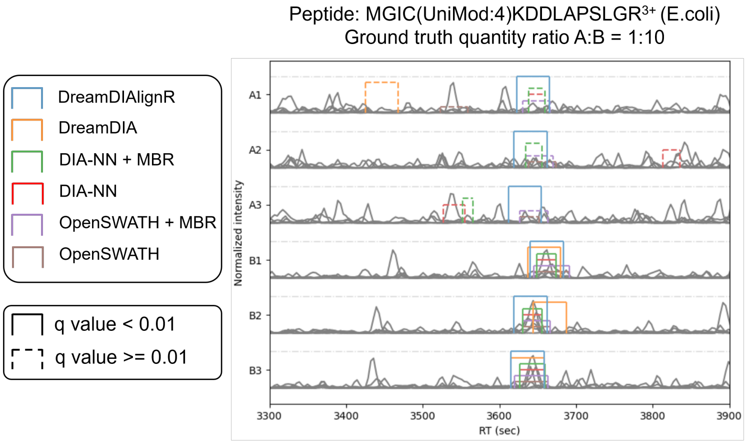

Quantification table

DreamDIAlignR	DreamDIA	DIA-NN + MBR	DIA-NN	OpenSWATH + MBR	OpenSWATH
1238	NaN (1607)	NaN (553)	NaN (553)	NaN (2413)	NaN (795)
1661	NaN (2626)	NaN (380)	NaN (200)	NaN (881)	NaN (1625)
2009	NaN (1506)	NaN (140)	NaN (1342)	NaN (1189)	NaN (516)
13784	14763	3224	3224	5876	5876
18637	11318	3088	3088	7560	7560
13453	13453	3370	3364	7555	7555

Figure S5. Example peak groups identified by different software tools on the LFQbench dataset. Peak groups identified for the following peptides: **a.** VAPTTGVVAK^{2+}, **b.** AYLHYIPK^{2+}, **c.** MHPDQDTLK^{2+}, **d.** MGIC(UniMod:4)KDDLAPSLGR^{3+}. Solid lines indicate peak groups with q-values below 1%, while dashed lines indicate those above 1%. Peptide quantities for each run, as reported by different software tools, are displayed alongside the corresponding chromatograms. Quantities in parentheses denote peak groups with q-values above 1%, indicating unreliable quantification. Dashed-line boxes or `NaN` values in the quantification table represent "not identified". Box heights vary solely to distinguish the different software tools for clearer visualization; they do not convey quantitative information.

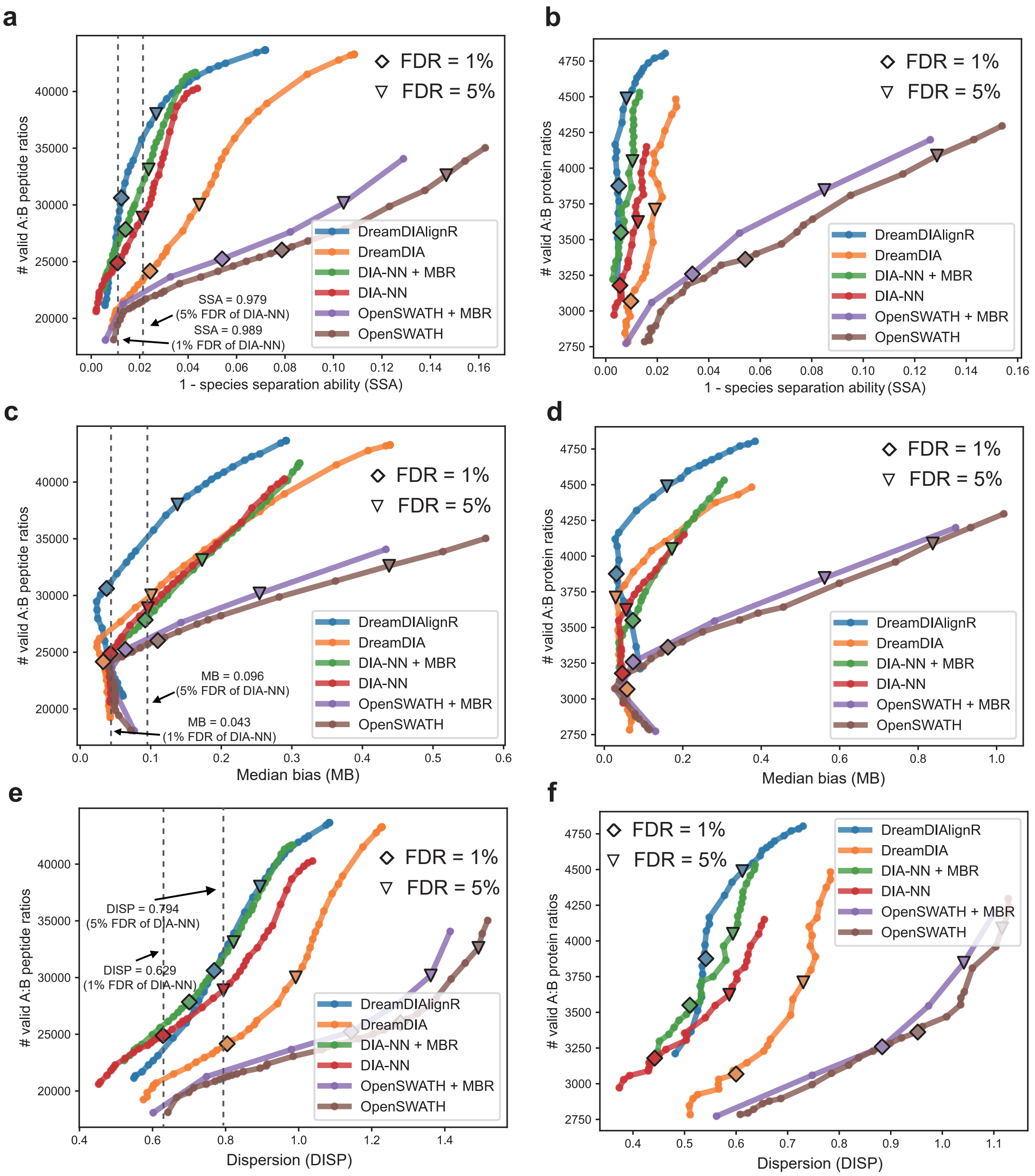

Figure S6. Comprehensive identification and quantification performance benchmark on LFQbench dataset. The number of valid Sample A to Sample B peptide ratios (**a, c, e**) and protein ratios (**b, d, f**) identified in total are plotted against the corresponding three quantification bias metrics including 1 - species separation ability (**a, b**), median bias (**c, d**) and dispersion (**e, f**) using a series of FDR thresholds. A ratio is considered valid if the peptide is identified in at least 1 run from both Sample A and Sample B. "◊" and "▽" denote the results at 1% and 5% peptide precursor FDR, respectively. Vertical dashed lines denote quantification cut-offs at 1% and 5% FDR, based on DIA-NN.

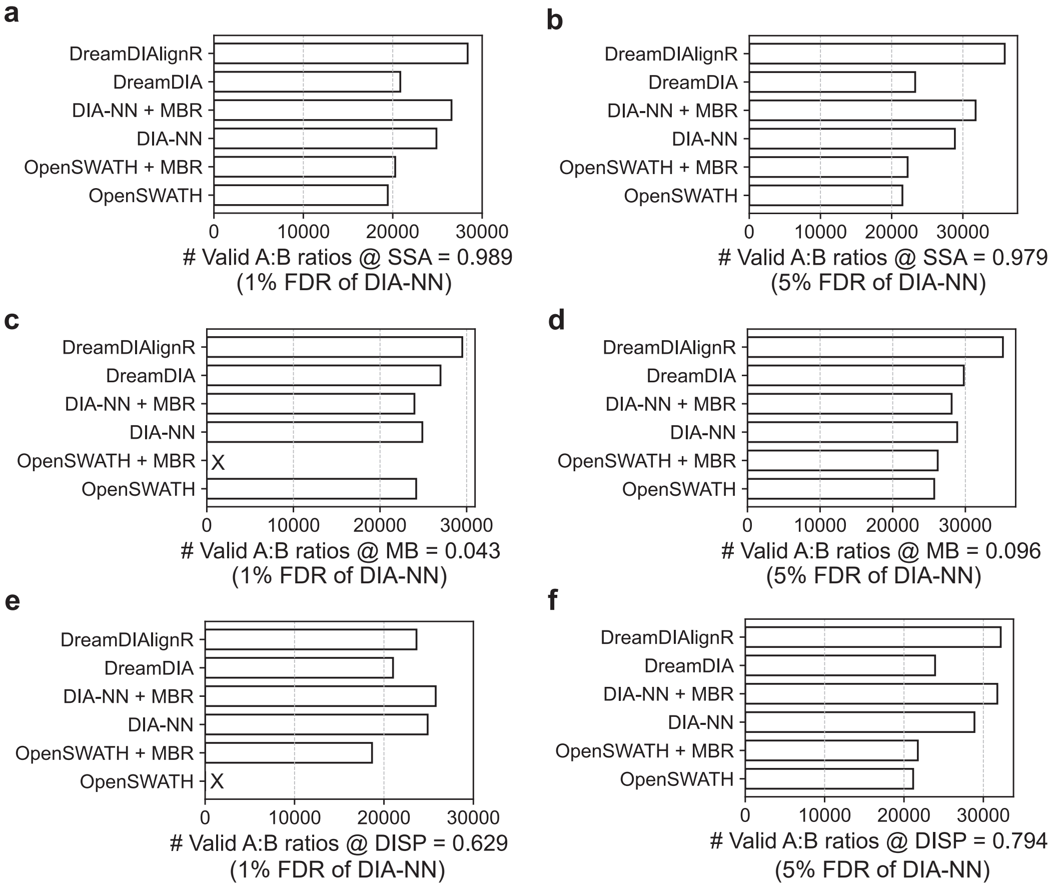

Figure S7. Number of valid peptide ratios identified by each tool at the benchmark quantification levels indicated in Supplementary Figure S6a, c, and e on the LFQbench dataset. Number of Sample A to Sample B peptide ratios identified at the following quantification levels, based on DIA-NN's peptide precursor FDR thresholds: **a.** SSA = 0.989 (1% FDR), **b.** SSA = 0.979 (5% FDR), **c.** MB = 0.043 (1% FDR), **d.** MB = 0.096 (5% FDR), **e.** DISP = 0.629 (1% FDR), **f.** DISP = 0.794 (5% FDR). "X" denotes that the software tool is unable to achieve the corresponding quantification metric.

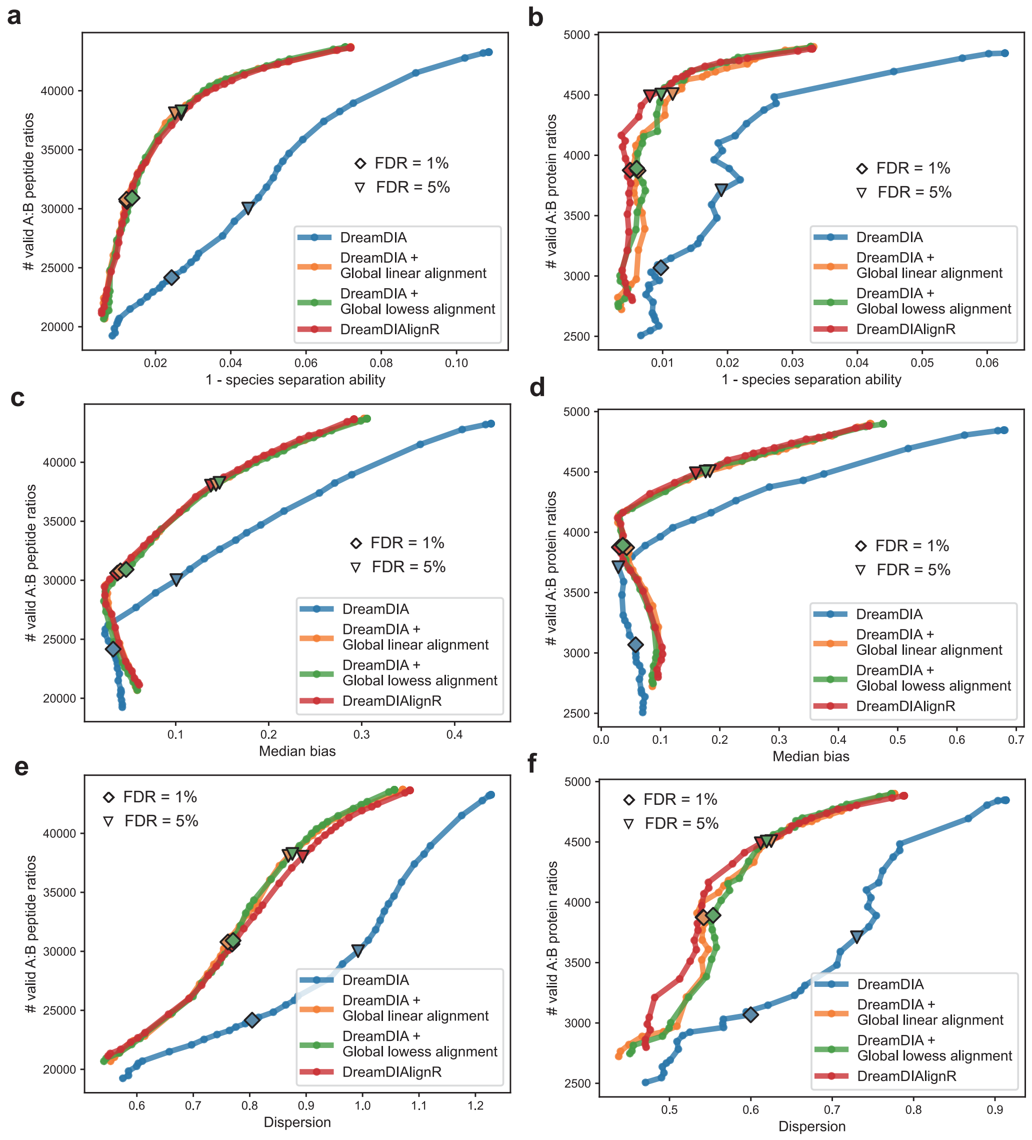

Figure S8. Identification and quantification performance benchmark of DreamDIAlignR using different signal alignment methods on the LFQbench dataset. The number of valid Sample A to Sample B peptide precursor ratios (**a, c, e**) and protein ratios (**b, d, f**) identified in total are plotted against the corresponding three quantification bias metrics including 1 - species separation ability, median bias and dispersion using a series of FDR thresholds.

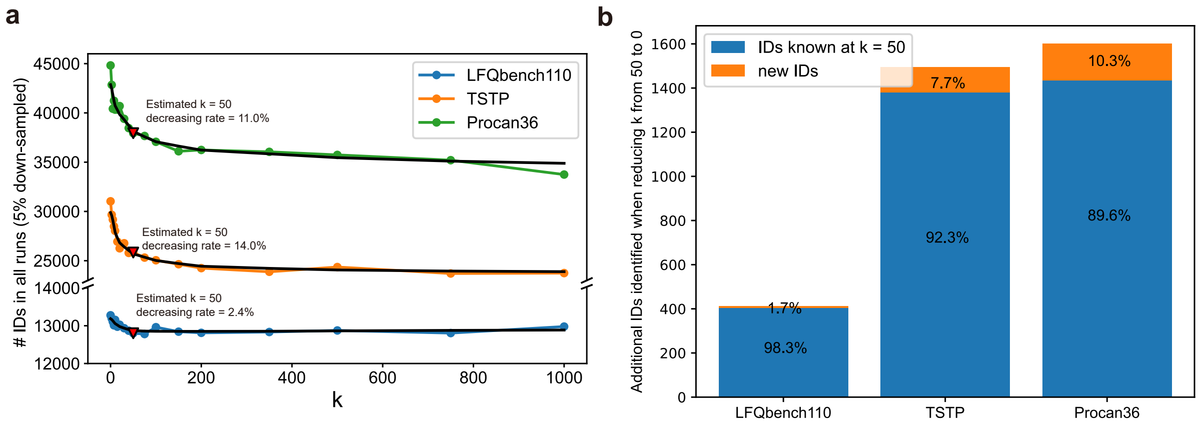

Figure S9. Estimating the weight decay parameter for different datasets. a. The total number of identified precursor IDs across all runs at 1% FDR, using a 5% down-sampled library, is plotted against the weight decay parameter "k". Black curves represent smoothed data points obtained using a Gaussian filter. Red triangles mark the estimated optimal k values. The decreasing rate is defined as the reduction in the number of identified precursors relative to the result at $k = 0$. **b.** Number of additional peptide identifications when reducing k from 50 to 0. Peptides already identified in at least one run at k = 50 are shown in blue, while newly identified peptides—those not detected in any run at k = 50—are shown in orange.

a

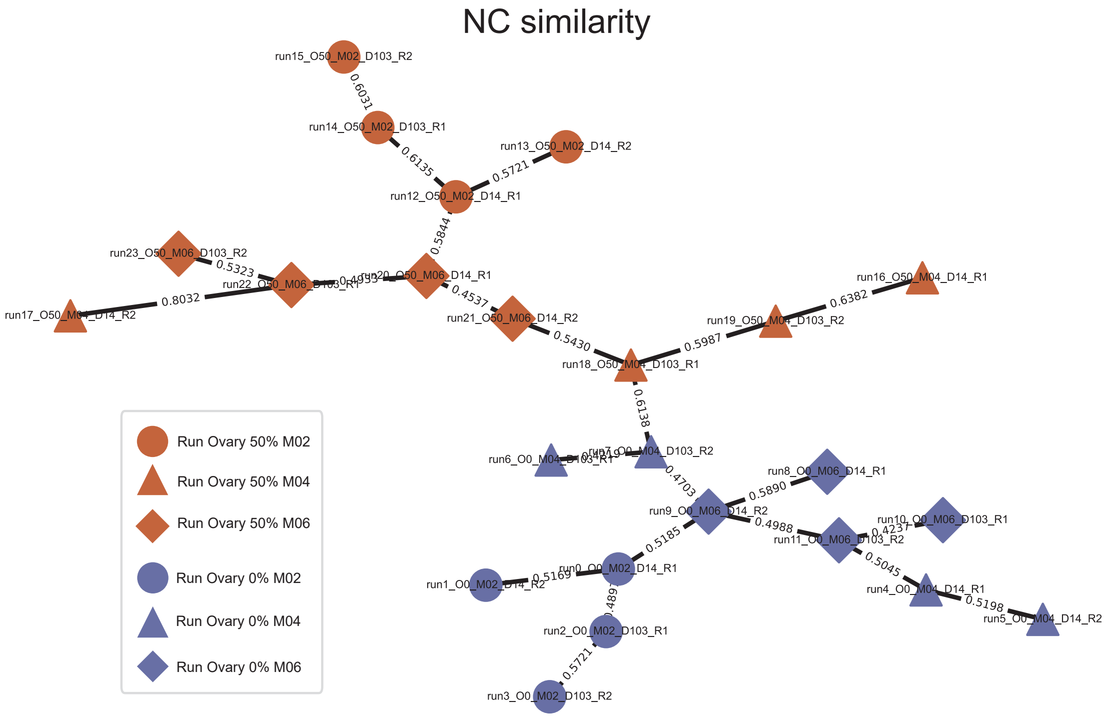
NC similarity
Run Ovary 50% M02
Run Ovary 50% M04
Run Ovary 50% M06
Run Ovary 0% M02
Run Ovary 0% M04
Run Ovary 0% M06

b

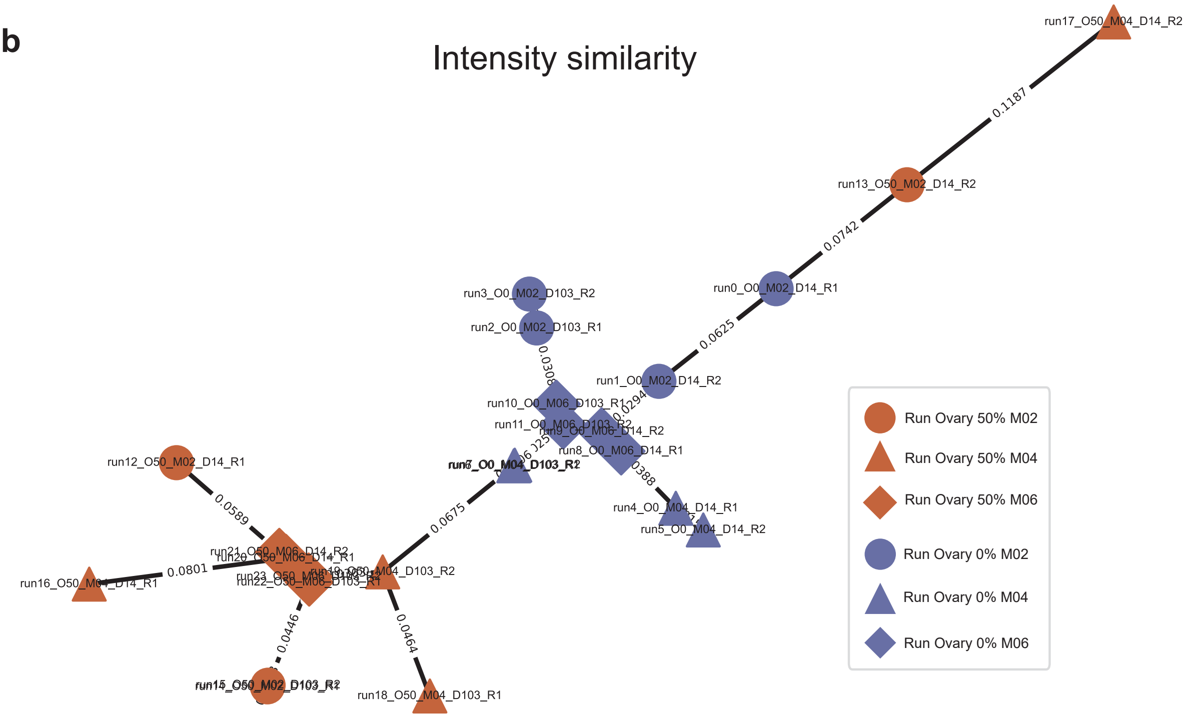
Intensity similarity
Run Ovary 50% M02
Run Ovary 50% M04
Run Ovary 50% M06
Run Ovary 0% M02
Run Ovary 0% M04
Run Ovary 0% M06

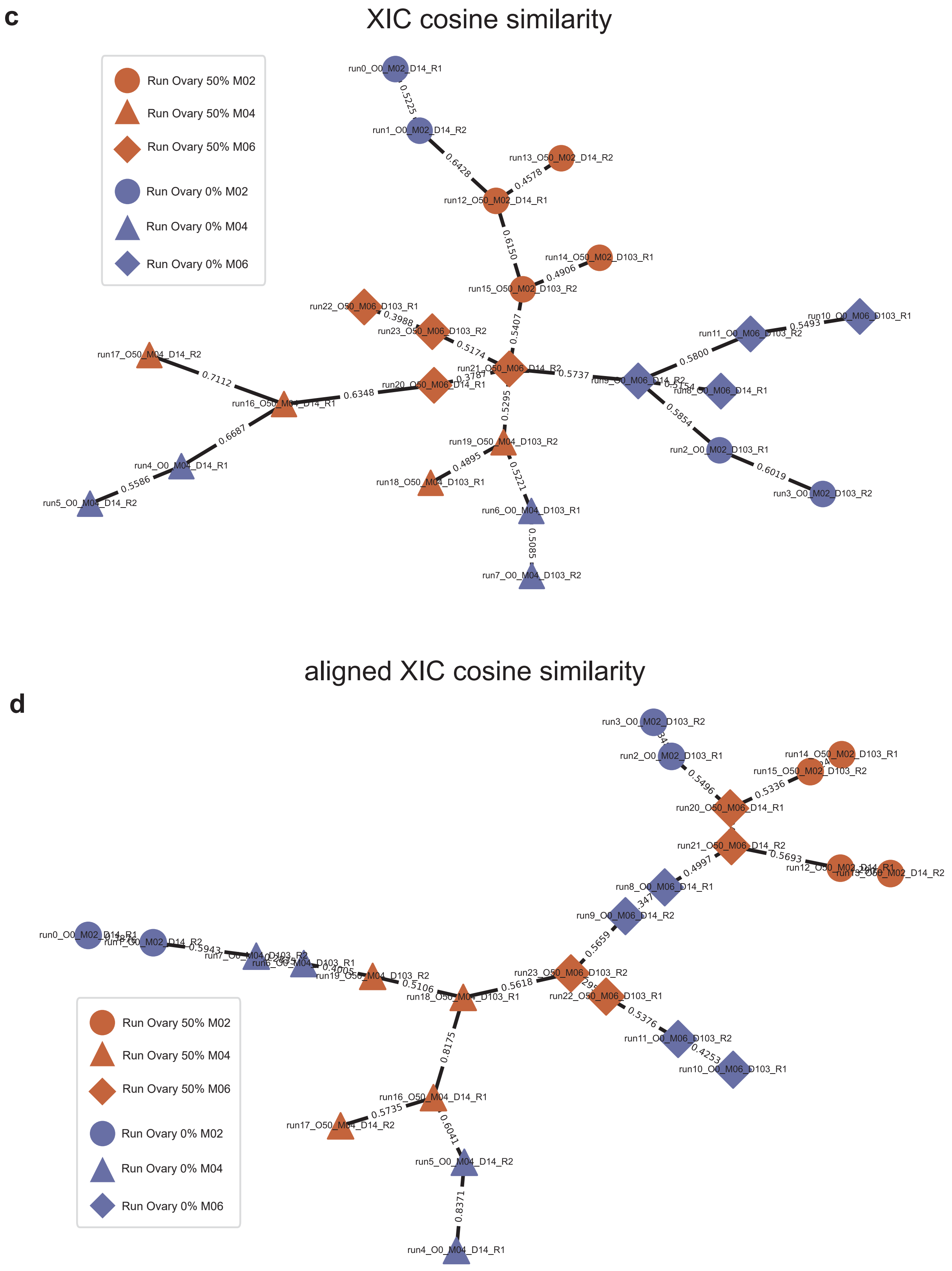

Figure S10. Sample minimum spanning trees constructed from global similarity matrices using different global similarity metrics on the TSTP dataset. a. NC similarity, **b.** Intensity similarity, **c.** XIC cosine similarity, **d.** Aligned XIC cosine similarity. Node colors indicate different sample types, and marker shapes represent the instruments used. Edge labels denote the distance between runs, calculated as $1 - \text{similarity}$.

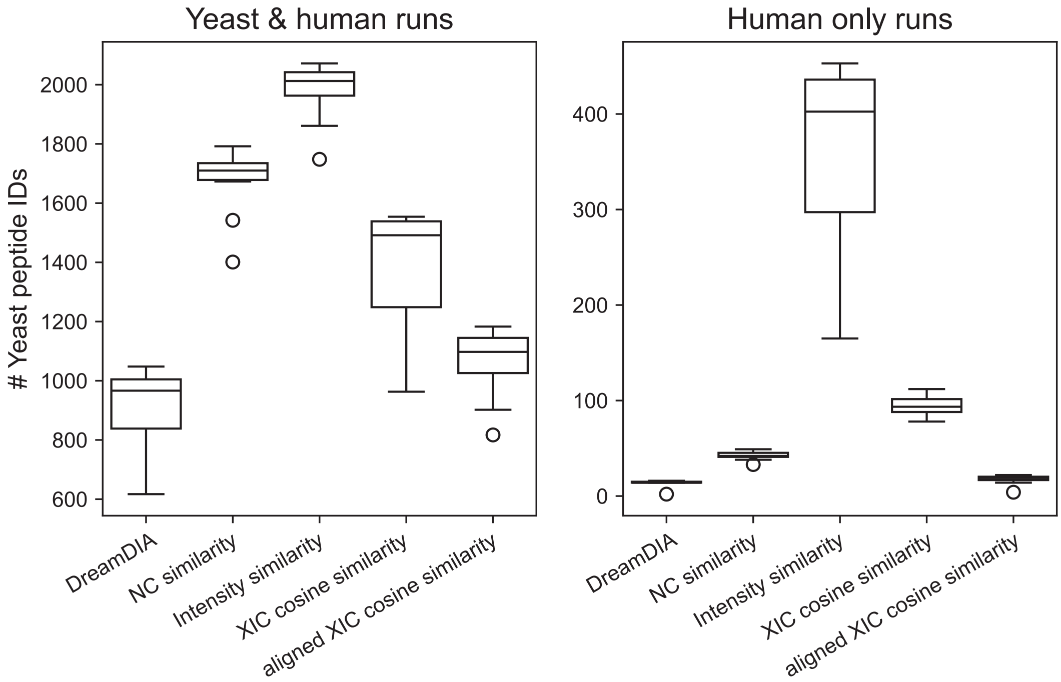

Figure S11. Number of yeast peptide IDs identified in human-yeast mixed runs and human-only runs using different global similarity metrics on the TSTP dataset. Boxplot elements: center line, median; boxes, interquartile range; whiskers, 1.5x interquartile range; points, outliers. Each box represents 12 independent experimental runs (n = 12).

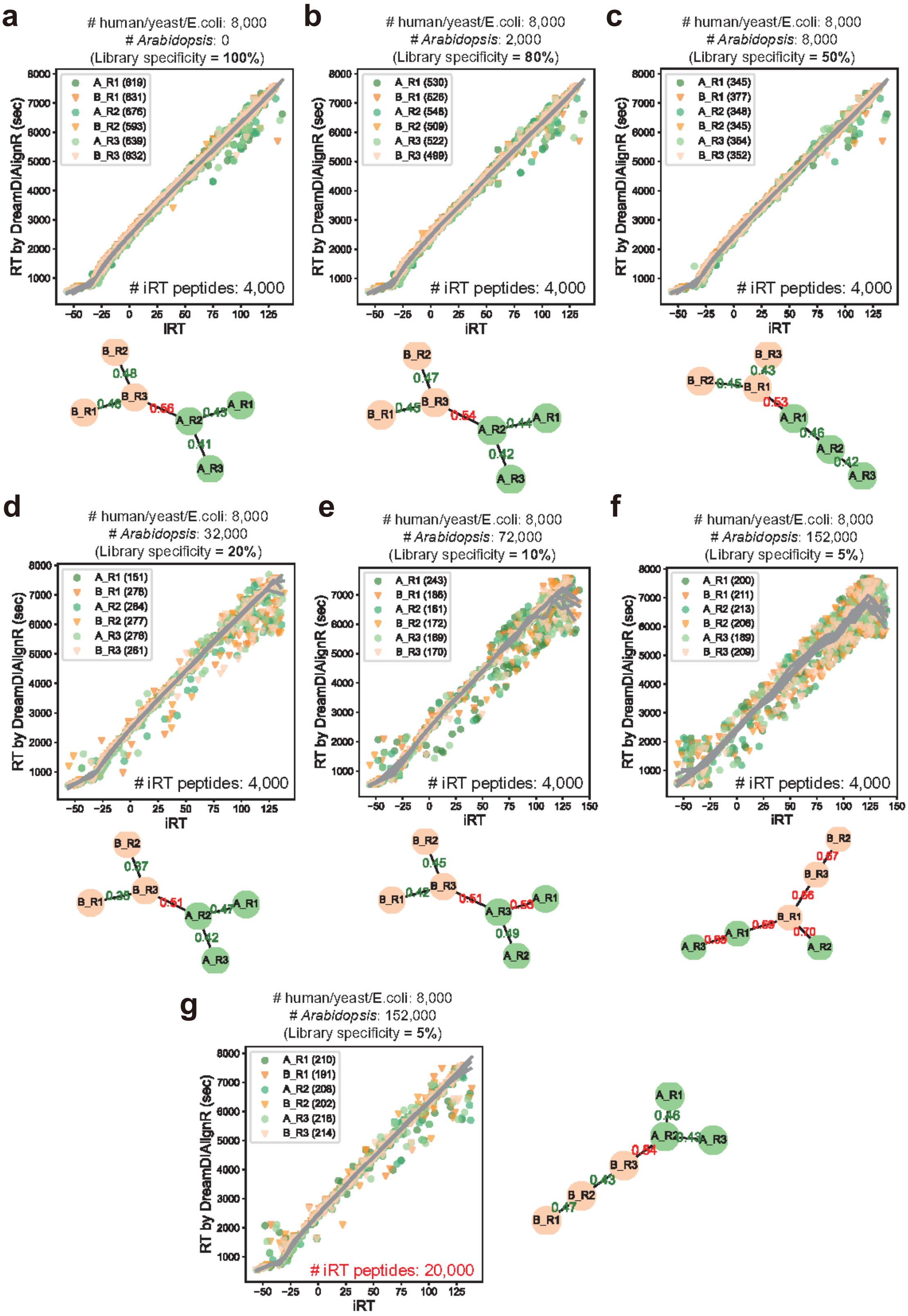

Figure S12. Effect of library specificity on global RT alignment performance on the LFQbench dataset. A series of libraries with varying specificity were generated by spiking different numbers of Arabidopsis peptides into the original library. **a–f.** RT normalization performance and the corresponding sample minimum spanning trees across different library specificities, using a fixed subset of 4,000 randomly selected iRT peptides. **g.** RT normalization performance and the corresponding minimum spanning tree when library specificity is 5% and 20,000 iRT peptides are used. Edges with distances greater than 0.5 are highlighted in red.

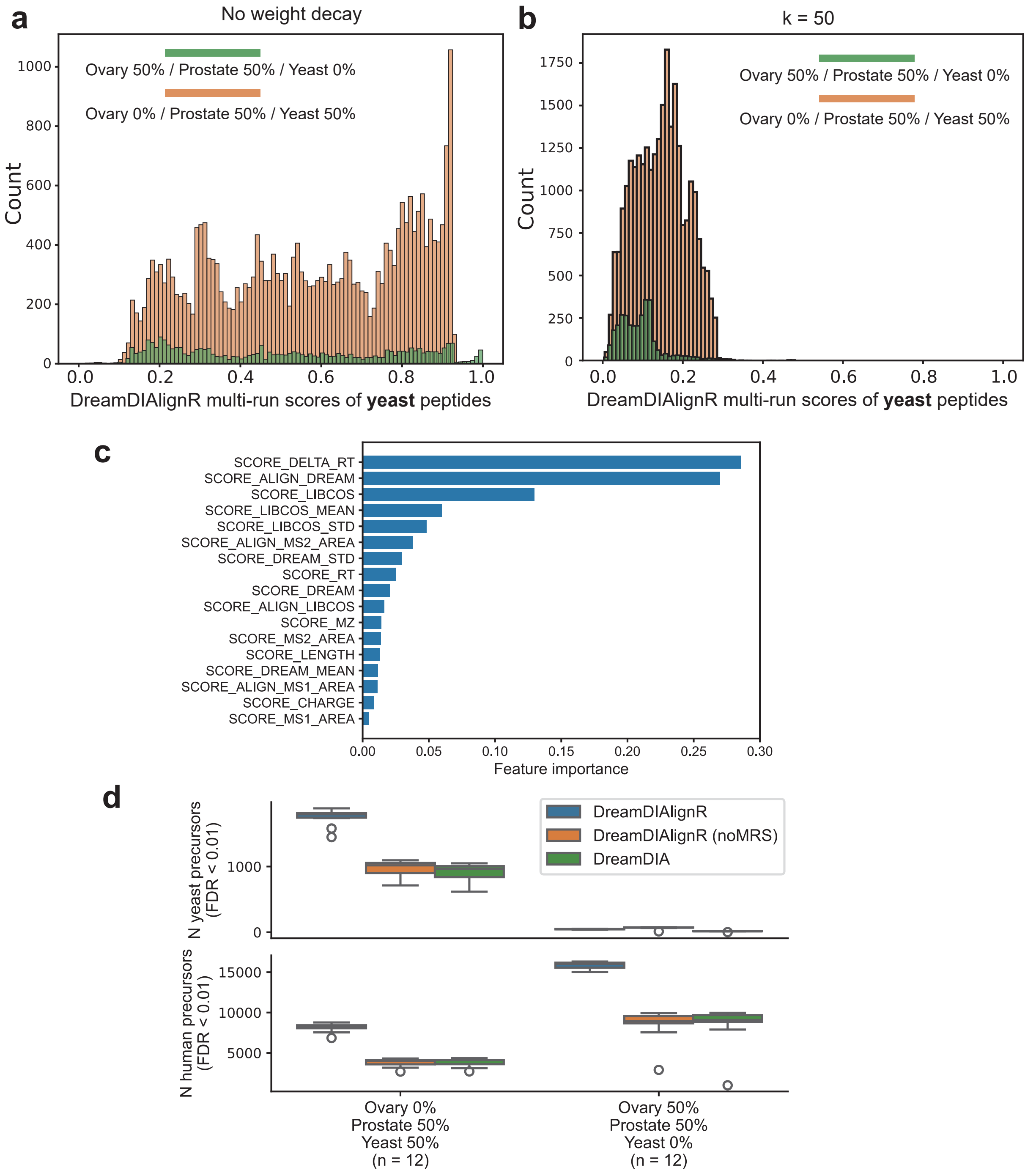

Figure S13. Effect of the weight decay parameter on multi-run score (MRS) calculation in the TSTP dataset. a, b. Distributions of the multi-run scores of yeast peptides in the two sample sets without (**a**) or with (**b**) weight decay. **c.** Feature importance of the statistical model when analyzing the TSTP dataset. The default XGBoost model was used. Scores with the prefix "SCORE_ALIGN" represent the multi-run scores calculated in DreamDIAlignR. **d.** The numbers of human and yeast peptide precursors identified by DreamDIA and DreamDIAlignR with and without the multi-run scores used by the discriminative model. Boxplot elements: center line, median; boxes, interquartile range; whiskers, 1.5x interquartile range; points, outliers. Each box represents 12 independent experimental runs (n = 12).

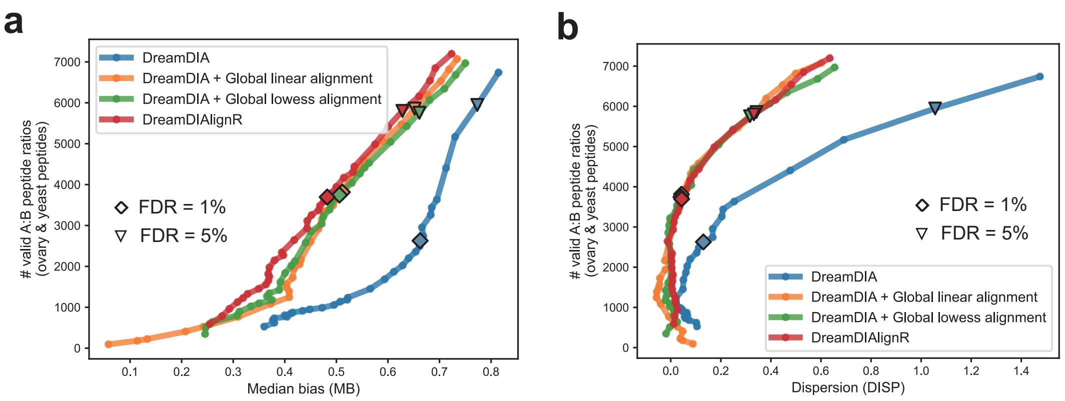

Figure S14. Performance of different signal alignment methods when analyzing highly heterogeneous dataset. The number of valid yeast and ovarian Sample A to Sample B peptide ratios is plotted against the corresponding quantification bias metrics, median bias (**a**) and dispersion (**b**) using a series of FDR thresholds. A peptide is named as a valid ratio only if it has been identified in at least 3 Sample A runs and 3 Sample B runs. The quantification bias metrics were calculated as suggested by LFQbench software package. "◊" and "▽" mean the results at 1% and 5% peptide precursor FDR respectively.

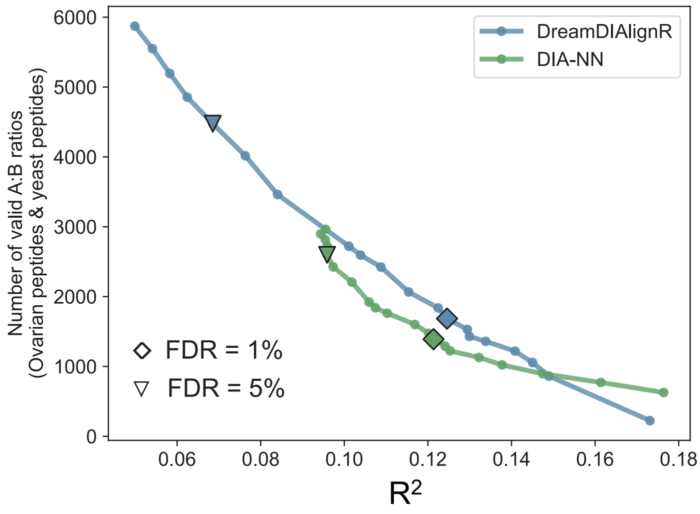

Figure S15. Performance of DreamDIAlignR and DIA-NN on Procan494 dataset. The number of valid peptide ratios is plotted against the corresponding quantification metric, the median of R^2, using a series of FDR thresholds. A valid ratio is defined as a peptide that has been identified in at least 10 runs for each sample. "◊" and "▽" denote the results at 1% and 5% peptide precursor FDR, respectively.

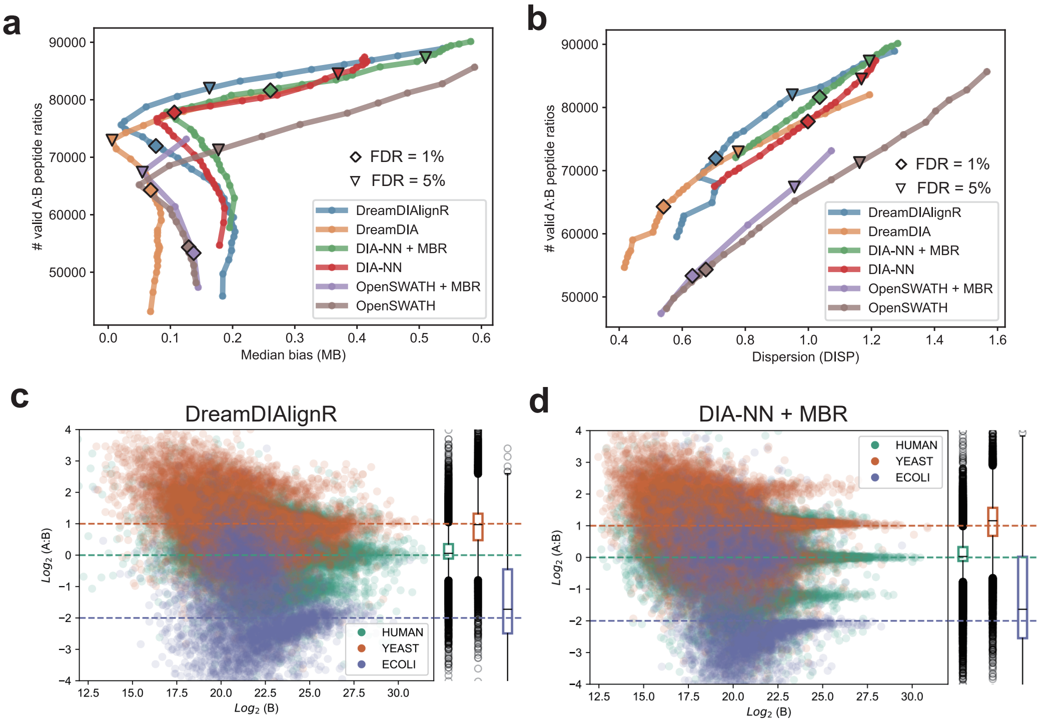

Figure S16. Identification and quantification performance benchmark on the Orbitrap LFQbench dataset. a, b. Number of valid peptide ratios (Sample A vs. Sample B), plotted against the corresponding median bias (MB; **a**) and dispersion (DISP; **b**) across a range of FDR thresholds. A ratio is considered valid if the peptide is identified in at least 3 runs from both Sample A and Sample B. Data points represent the mean MB and DISP across yeast and *E. coli* peptides. "$\Diamond$" and "$\bigtriangledown$" indicate results at 1% and 5% precursor FDR, respectively. **c, d.** Log-transformed Sample A to Sample B ratios plotted against $\log_2$(B) concentrations for different species, comparing DreamDIAlignR and DIA-NN. Results were filtered to include the same number of precursors (81,645), based on DIA-NN's 1% precursor FDR. Number of peptide precursors: DreamDIAlignR ($N_{\mathrm{human}} = 51319$, $N_{\mathrm{yeast}} = 24726$, $N_{\mathrm{E.coli}} = 5600$); DIA-NN ($N_{\mathrm{human}} = 51079$, $N_{\mathrm{yeast}} = 25306$, $N_{\mathrm{E.coli}} = 5260$). Colored dashed lines indicate the log-transformed ground truth Sample A to Sample B ratios (Human: 1:1; Yeast: 2:1; *E. coli*: 1:4). Boxplot elements: center line, median; boxes, interquartile range; whiskers, 1.5× interquartile range; points, outliers.

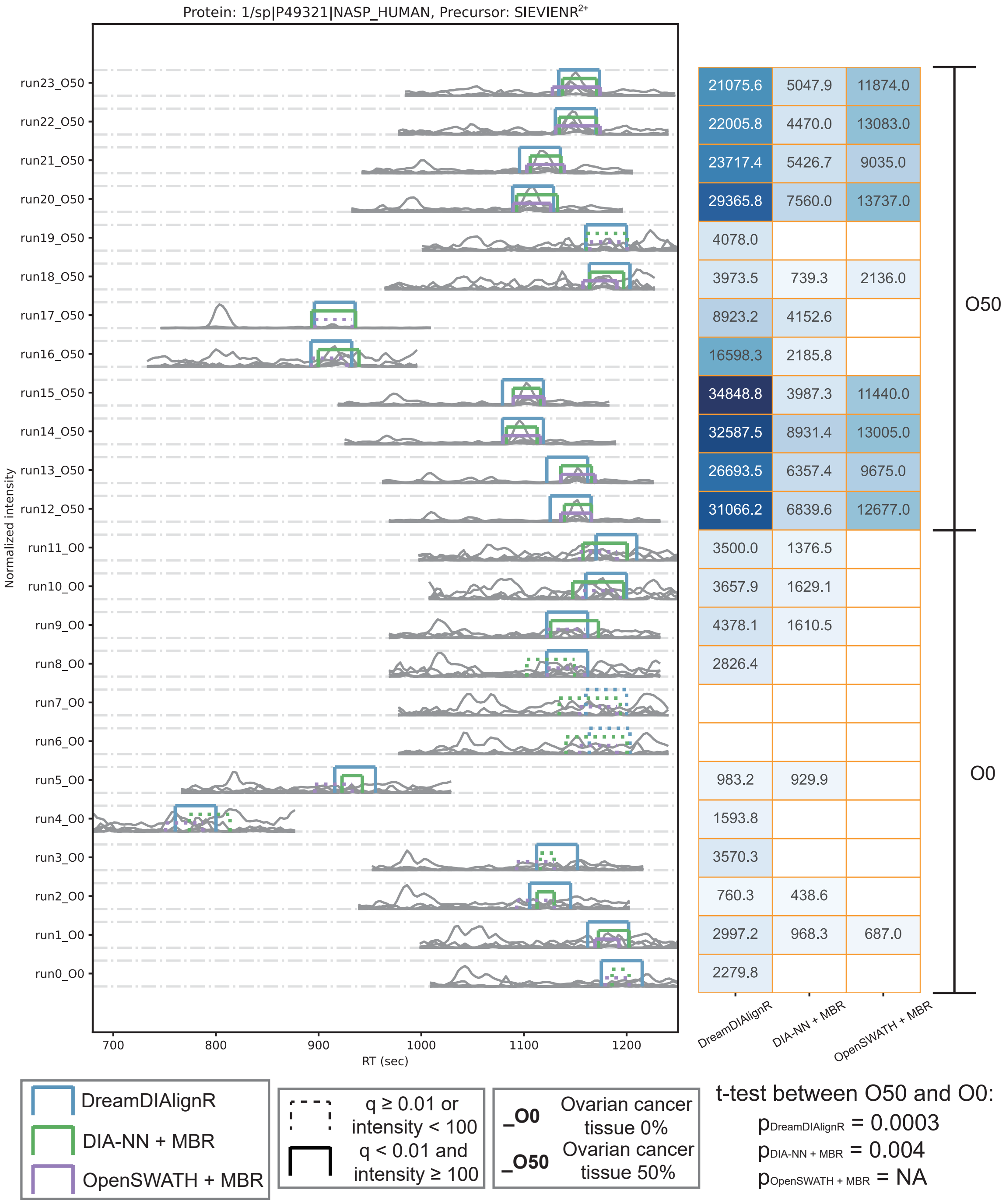

Figure S17. Chromatographic peaks of the precursor SIEVIENR^{2+} from the protein NASP identified by three different software tools and their corresponding quantifications across 24 runs in the TSTP dataset. Colored boxes represent the peak boundaries assigned by each software tool. Solid lines indicate valid identifications, defined as having a q-value below 1% and intensity above 100, whereas dashed lines denote invalid identifications. Adjacent run pairs (e.g., even and odd runs such as Run0 and Run1, Run2 and Run3, Run4 and Run5) are technical replicates acquired under identical experimental conditions. Two-tailed t-tests were conducted between the precursor intensities of the “O50” and the “O0” groups without intensity imputation or normalization, with intensities in the “O0” group multiplied by 2 for fair comparison.

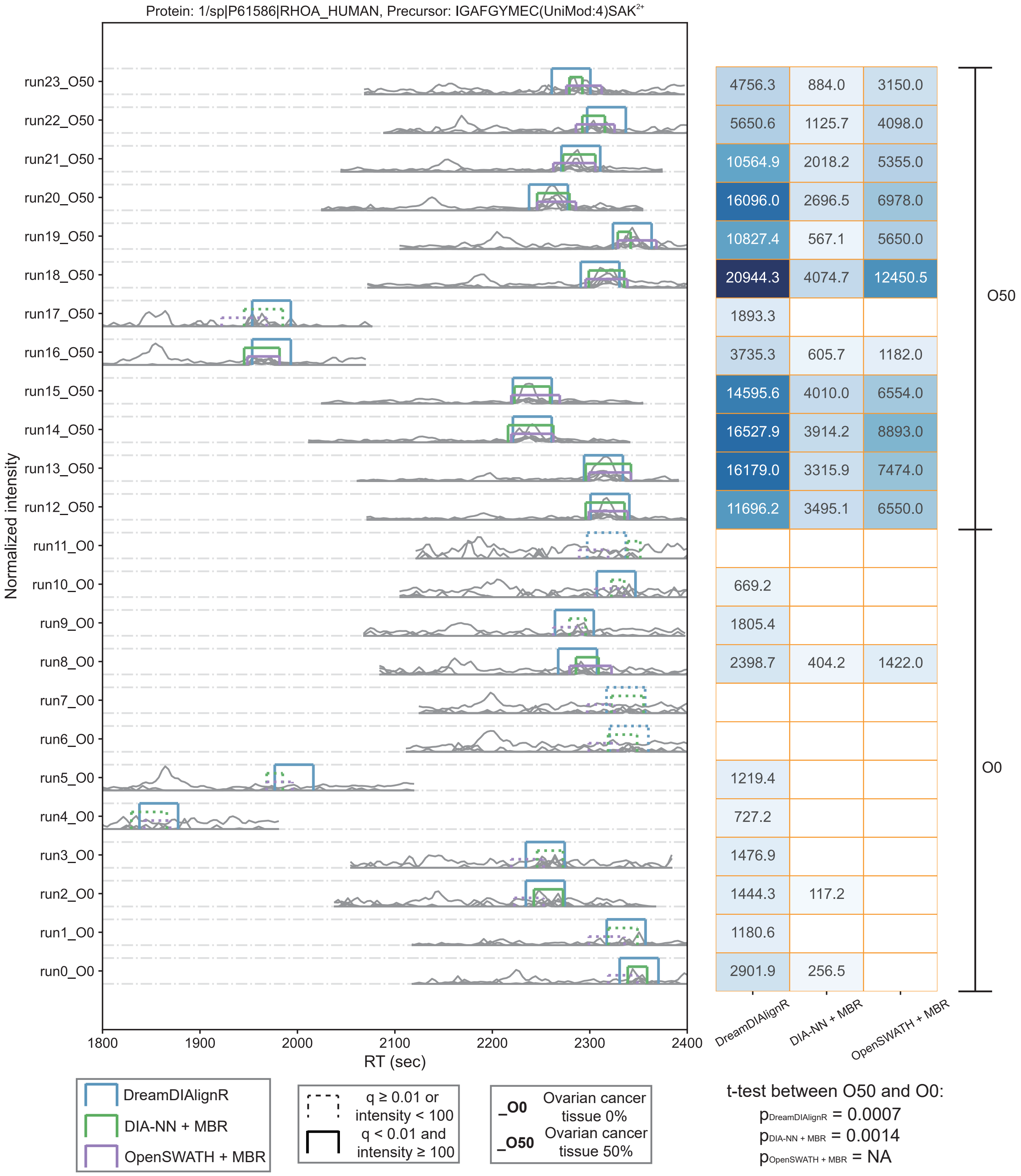

Figure S18. Chromatographic peaks of the precursor IGAFGYMEC(UniMod:4)SAK^{2+} from the protein RHOA identified by three different software tools and their corresponding quantifications across 24 runs in the TSTP dataset. Colored boxes represent the peak boundaries assigned by each software tool. Solid lines indicate valid identifications, defined as having a q-value below 1% and intensity above 100, whereas dashed lines denote invalid identifications. Adjacent run pairs (e.g., even and odd runs such as Run0 and Run1, Run2 and Run3, Run4 and Run5) are technical replicates acquired under identical experimental conditions. Two-tailed t-tests were conducted between the precursor intensities of the "O50" and the "O0" groups without intensity imputation or normalization, with intensities in the "O0" group multiplied by 2 for fair comparison.

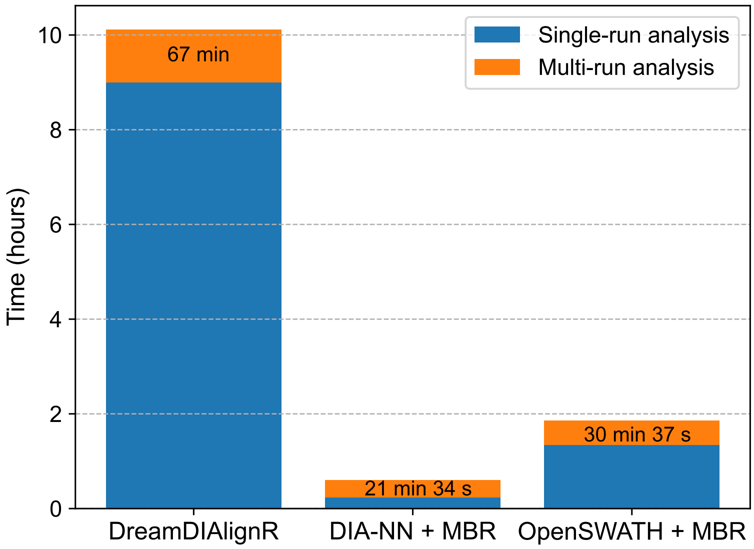

Figure S19. Speed benchmark of the software tools. Six runs from the Orbitrap LFQbench dataset were used in this experiment. All software tools were limited to 16 CPU cores and 64 GB of memory. DreamDIAlignR was additionally assigned a single GPU core as required. The multi-run analysis time of DreamDIAlignR was calculated by subtracting the single-run analysis time of DreamDIA from the total runtime of DreamDIAlignR. For DIA-NN, the multi-run analysis time corresponds to the "second-pass" processing step indicated in its runtime log. For OpenSWATH, the multi-run analysis time reflects the runtime of DIAlignR applied to OpenSWATH's single-run analysis results.

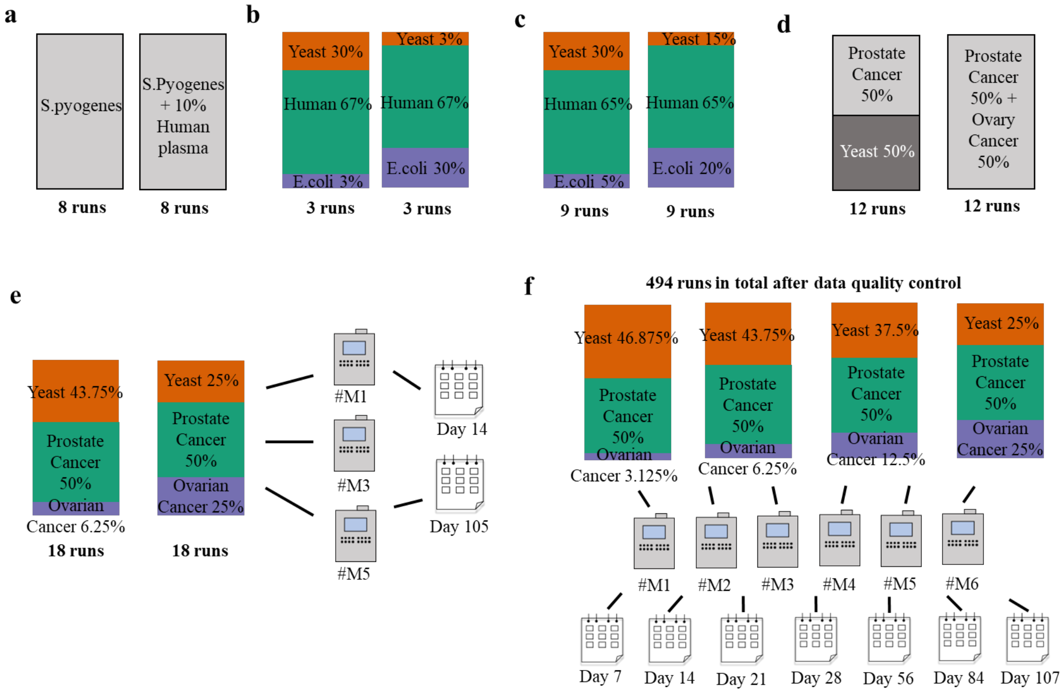

Figure S20. Schematic overview of the datasets analyzed in this study. a. S.pyogenes dataset. **b.** LFQbench HYE110 dataset. **c.** Orbitrap LFQbench dataset. **d.** TSTP dataset. **e.** Procan36 dataset. **f.** Procan494 dataset.
