# Supplementary material for: Scoring information integration with statistical quality control enhanced cross-run analysis of data-independent acquisition proteomics data
Source: Commun Chem. 2025 Nov 20;8:364. doi: 10.1038/s42004-025-01734-5 (PMC12635196; doi:10.1038/s42004-025-01734-5)
Supplement: Supplementary file 1 — Transparent Peer Review file [file 42004_2025_1734_MOESM1_ESM.pdf]

This file contains all reviewer reports in order by version, followed by all author rebuttals in order by version.

nature portfolio

Peer Review File

Scoring information integration with statistical quality control enhanced cross-run analysis of data-independent acquisition proteomics data

Corresponding Author: Dr Hannes Röst

Version 0:

Reviewer comments:

Reviewer #1

(Remarks to the Author)
The authors present DreamDIAAlignR, that essentially performs MBR in an integrative approach, taking many more features into account such as peptide elution behavior. I especially appreciate the availability of the code (under apache 2.0) and the final evaluation where the impact on real analysis and biological conclusions is compared between tools. I would suggest a major revision where some of the concerns outlined below are addressed.

Major concerns

1. All acquisition, as far as I could see, are performed on either a SCIEX TripleTOF 5600 or 6600. Could the authors please also evaluate their tool on instruments from different vendors/DIA strategies? I can recommend PXD028735

2. The authors introduce one of their own metric called Overall Performance Distance (OPD). I do not find the case for this metric very convincing as the metric is not extensively explained or evaluated. Furthermore, I expect the combined metrics for this meta-metric are likely not independent from each other; if they are not complementary I do not see how this metric can offer much advantage. Have the authors tried plotting the data in 3 dimensions (so selecting 4 out of 3) or even giving a scatter plot a 4th dimension through color. It seems that the conclusions made in the manuscript do not hinge on this metric.

3. The percentages mentioned in the manuscript before and after MBR seem very high between the tools, but one needs to read very carefully to discover the percentages in within tools and not between them. This might cause confusion for the reader and I would suggest to rewrite these parts to make them clearer.

4. The conclusions of the manuscript would be even stronger if the authors performed an entrapment experiment. While they have a two-proteome experiment, I would also suggest to add random (or other species) proteomes that are not part of any of the samples.

5. Could the authors go a bit more in-depth for the identification differences? I would like to see what the overlap is between identifications of the different tools. Also for the unique ones of DreamDIAlignR, could the authors inspect some of the MS2 XICs?

Minor concerns

-

Reviewer #2

(Remarks to the Author)
This study presents DreamDIAlignR, a novel cross-run analysis tool that integrates deep learning-based peak scoring (DreamDIA) with dynamic programming alignment (DIAlignR) and statistical quality control to address challenges in peptide identification and quantification of DIA proteomics data. The work claims improvements in cross-run identification consistency and quantification accuracy, comparing with current open-source DIA data interpretation software. However, there are still several major points need to be addressed before publication.
Major Concerns and Suggestions

1. The authors mentioned that a subset of peptides from the spectral library is initially selected as anchor peptides to build RT alignment models. Please further explain the principle of choosing these anchor peptides as it is vital for RT alignment. Also, we are curious that would the performance of RT alignment be negatively influenced, if these selected anchor peptides are not available in the chromatogram spectra.
2. The authors use NC similarity to calculate multi-run scores, which solely rely on number of common iRT peptides between different runs. Intuitively, this approach seems to ignore most information in the chromatogram spectra. The authors should compare the performance of NC similarity with different method, which include other key information, such as relative intensity of peeks or even embed the whole aligned chromatogram spectra into a representative vector for similarity communication.
3. While the weight decay parameter (k=50) effectively reduces false positives in the tested datasets, its universality across diverse experimental setups remains unclear. A sensitivity analysis or adaptive parameter selection strategy would strengthen the method’s robustness.

Reviewer #3

(Remarks to the Author)
The manuscript “COMMSCHEM-25-0094-T” describes and benchmarks DreamDIAlignR, a cross-run peptide-centric tool for data-independent acquisition (DIA) analyses that integrates peptide elution behavior across runs with a deep learning peak identifier and signal alignment algorithm for consistent peak picking and FDR-controlled scoring. The integration of multi-run alignment techniques and statistical validation addresses known limitations in current DIA analyses, improving reproducibility and quantification performance. The manuscript provides an open-source implementation and publicly available datasets which enhances reproducibility and transparency. The authors systematically compare their method with established workflows using multiple datasets. The following recommendations are provided to improve the manuscript:
1. It would be helpful for the readers to include a brief description of the pre-trained LSTM model, the first time is mentioned in the manuscript, and how it was trained.
2. Fig. 2 a-c, clarify what the gray and white boxes on the axis are representing.
3. Fig. 2, consider if this representation of peptide charge state is commonly used: ADGQTVSGGSILYR/3.
4. Fig. 5 could be simplified by comparing only the results using MBR. It is clear that MBR mostly helps for each tool. Make it easier to see how your method is performing better compared to other tools. In the end, what the users want to know is which software tool provides more correct IDs and more accurate quantitation.
5. Fig. 6 d, the boxes inside the circles are hard to see, consider increasing the size and using a different symbol for each the tool.
6. Software information, specify the programming language(s) of the package.
7. Consider adding a brief discussion comparing the running times of the tools.

Version 1:

Reviewer comments:

Reviewer #1

(Remarks to the Author)
The authors have substantially improved their manuscript and I have no further comments.

Reviewer #2

(Remarks to the Author)
The reviewers have addressed all my previous concerns. I would like to suggest to publish the work as is.

Reviewer #3

(Remarks to the Author)
The authors have addressed the reviewers' concerns and suggestions.

Open Access This Peer Review File is licensed under a Creative Commons Attribution 4.0 International License, which permits use, sharing, adaptation, distribution and reproduction in any medium or format, as long as you give appropriate credit to the original author(s) and the source, provide a link to the Creative Commons license, and indicate if changes were made.
In cases where reviewers are anonymous, credit should be given to 'Anonymous Referee' and the source.
The images or other third party material in this Peer Review File are included in the article's Creative Commons license, unless indicated otherwise in a credit line to the material. If material is not included in the article's Creative Commons license and your intended use is not permitted by statutory regulation or exceeds the permitted use, you will need to obtain permission directly from the copyright holder.
To view a copy of this license, visit https://creativecommons.org/licenses/by/4.0/

Response to reviewers

Reviewer #1

The authors present DreamDIAlignR, that essentially performs MBR in an integrative approach, taking many more features into account such as peptide elution behavior. I especially appreciate the availability of the code (under apache 2.0) and the final evaluation where the impact on real analysis and biological conclusions is compared between tools. I would suggest a major revision where some of the concerns outlined below are addressed.

1. All acquisition, as far as I could see, are performed on either a SCIEX TripleTOF 5600 or 6600. Could the authors please also evaluate their tool on instruments from different vendors/DIA strategies? I can recommend PXD028735.

We thank the reviewer for this valuable suggestion. In the revised manuscript, we have included a new benchmark experiment using the Orbitrap All-Ion Fragmentation (AIF) LFQbench dataset (PXD028735; Supplementary Figure S16) to evaluate the performance of DreamDIAlignR on data acquired from a different vendor and with a different DIA strategy. The results demonstrate that DreamDIAlignR maintains strong identification and quantification performance, supporting its robustness across diverse instrument platforms and acquisition methods.

2. The authors introduce one of their own metric called Overall Performance Distance (OPD). I do not find the case for this metric very convincing as the metric is not extensively explained or evaluated. Furthermore, I expect the combined metrics for this meta-metric are likely not independent from each other; if they are not complementary I do not see how this metric can offer much advantage. Have the authors tried plotting the data in 3 dimensions (so selecting 4 out of 3) or even giving a scatter plot a 4th dimension through color. It seems that the conclusions made in the manuscript do not hinge on this metric.

We thank the reviewer for raising this important point. The OPD metric was initially introduced to quantify the overall performance improvement brought by DreamDIAlignR in a way that avoids potential biases from selecting overly optimized FDR thresholds. However, we agree that the metric may be unintuitive for readers and that its added value is limited, particularly given the potential dependence among the contributing quantification metrics.

We also explored the reviewer's suggestion of visualizing the data using a 3D scatter plot, and

even considered adding a fourth dimension via color. However, we found that the data points were often clustered too closely together, making interpretation difficult.

In light of these concerns, we have removed the OPD metric in the revised manuscript and replaced it with a more transparent and intuitive evaluation approach. Specifically, we now compare the number of identifications across software tools at matched quantification accuracy levels (Figure 3f, Figure 5d and Supplementary Figure S7). This is done by drawing cutoff lines at widely accepted FDR thresholds using multiple quantification metrics. To avoid the appearance of bias—specifically, selecting FDR thresholds that might favor DreamDIAlignR—we use DIA-NN's FDR thresholds as reference points for all software comparisons. We believe this revised approach more clearly illustrates the performance advantages of DreamDIAlignR while remaining easy to interpret and free from assumptions about metric independence.

3. The percentages mentioned in the manuscript before and after MBR seem very high between the tools, but one needs to read very carefully to discover the percentages in within tools and not between them. This might cause confusion for the reader and I would suggest to rewrite these parts to make them clearer.

We thank the reviewer for this valuable feedback. In the revised manuscript, we have rewritten the benchmarking section to clearly distinguish between two types of comparisons: (1) internal comparisons within each tool before and after applying MBR, and (2) overall performance comparisons across different software tools. These two contexts are now presented in separate paragraphs to avoid confusion and improve readability.

One of the key contributions of DreamDIAlignR is that it is not simply another DIA analysis tool, but rather a novel and generalizable MBR approach that can, in principle, be integrated with any existing DIA software. Therefore, benchmarking its effectiveness as an MBR strategy—separately from overall tool performance—is an important aspect of our work.

4. The conclusions of the manuscript would be even stronger if the authors performed an entrapment experiment. While they have a two-proteome experiment, I would also suggest to add random (or other species) proteomes that are not part of any of the samples.

We thank the reviewer for the insightful suggestion. In the revised manuscript, we now explicitly clarify that the previously described two-proteome test is indeed a form of entrapment experiment. Notably, this test serves as an even more stringent evaluation than conventional entrapment using peptides from unrelated species, as it challenges the MBR algorithm not only

to correctly identify peptides but also to assign them to the appropriate runs—thereby avoiding false alignments across samples. This makes the task significantly more demanding for any DIA analysis tool employing MBR.

Building on the reviewer's suggestion, we also conducted a complementary entrapment analysis by spiking Arabidopsis peptides into the LFQbench dataset (Figure 3e). These peptides are not present in any runs, providing a robust negative control. The results show that while DIA-NN's MBR inflated the FDR by approximately threefold, and OpenSWATH's MBR produced overly conservative estimates due to hard filtering of low-confidence identifications, DreamDIAlignR maintained a well-calibrated FDR closely matching the actual two-species FDR. This demonstrates that DreamDIAlignR effectively controls false positives and offers robust and reliable FDR estimation. Notably, even when compared against DIA-NN's benchmark using an inflated FDR, DreamDIAlignR still achieved superior performance.

5. Could the authors go a bit more in-depth for the identification differences? I would like to see what the overlap is between identifications of the different tools. Also for the unique ones of DreamDIAlignR, could the authors inspect some of the MS2 XICs?

We appreciate the reviewer's suggestion to further investigate the identification differences across tools. In the revised manuscript, we have added Venn diagrams illustrating the overlap of identified peptides among different software tools (Figure 3d, Figure 5e, Figure 6b, Supplementary Figure S3d, and S4).

Additionally, we inspected the MS2 XICs and provided representative chromatograms for several peptides identified by different tools, now shown in Supplementary Figures S5, S17, and S18. In these figures, peaks marked by solid lines indicate valid identifications. Peptides surrounded by solid blue lines and dashed/missing lines in other colors represent identifications unique to DreamDIAlignR.

It is worth noting that peptides uniquely identified by DreamDIAlignR across all runs—meaning they were not identified in any run by any other tool—are rare, and not the main focus of what DreamDIAlignR aims to improve. Instead, DreamDIAlignR typically recovers peptides that are partially identified by other tools—i.e., those found in some runs but missed or wrongly identified in others—and ensures more consistent and accurate identification across runs. This is a key advantage of the match-between-runs (MBR) strategy.

By presenting both overlap analyses and visual comparisons of MS2 XICs, we aim to provide a clearer picture of the consistency and accuracy improvements enabled by DreamDIAlignR.

Reviewer #2

This study presents DreamDIAlignR, a novel cross-run analysis tool that integrates deep learning-based peak scoring (DreamDIA) with dynamic programming alignment (DIAlignR) and statistical quality control to address challenges in peptide identification and quantification of DIA proteomics data. The work claims improvements in cross-run identification consistency and quantification accuracy, comparing with current open-source DIA data interpretation software. However, there are still several major points need to be addressed before publication.

1. The authors mentioned that a subset of peptides from the spectral library is initially selected as anchor peptides to build RT alignment models. Please further explain the principle of choosing these anchor peptides as it is vital for RT alignment. Also, we are curious that would the performance of RT alignment be negatively influenced, if these selected anchor peptides are not available in the chromatogram spectra.

We thank the reviewer for raising this important point. Retention time (RT) alignment is indeed a critical component of DreamDIAlignR's workflow. To ensure robust RT calibration (iRT to RT in single runs) and RT alignment (global RT alignment between runs), DreamDIAlignR performs stratified random sampling of anchor peptides from the spectral library. Specifically, the iRT range (e.g., -50 to 250) is divided into equal-width bins (layers), and peptides are randomly selected from each layer to ensure full iRT coverage. Typically, 400 peptides are sampled within each layer, with a 20% oversampling in the first and last layers to improve model accuracy at the edges. This approach does not rely on spike-in standards and enables consistent performance across datasets with different acquisition settings. A brief description of this method has been added to the Methods section in the revised manuscript.

Regarding the concern about missing anchor peptides in the chromatographic data: yes, the quality of RT alignment can degrade if a significant portion of the selected peptides are absent from the spectra. To evaluate this, we conducted an entrapment experiment (described in Supplementary Figure S12 of the revised manuscript). We added various amounts of *Arabidopsis* peptides to a spectral library built for the LFQbench dataset (which contains only human, yeast, and E. coli peptides), thereby reducing library specificity from 100% down to 5%.

As expected, when the proportion of true peptides in the library dropped, RT alignment performance declined. At 5% specificity (i.e., only ~200 real peptides among 4,000), the alignment models became unreliable, and the global alignment across runs—reflected by the

minimum spanning tree (MST) used in multi-run scoring—was inaccurate. However, when we increased the number of anchor peptides sampled (via the --n_irts argument) from 4,000 to 20,000, around 800 of them were possibly real, and both RT calibration and global alignment performance recovered.

This demonstrates that as long as a reasonable number of true peptides are sampled, DreamDIAlignR's peak scoring and alignment remain robust—even in the presence of a large number of false entries. In practice, a library with only 5% specificity is already an extreme case, and DreamDIAlignR still manages to build reliable alignment models under such condition.

2. The authors use NC similarity to calculate multi-run scores, which solely rely on number of common iRT peptides between different runs. Intuitively, this approach seems to ignore most information in the chromatogram spectra. The authors should compare the performance of NC similarity with different method, which include other key information, such as relative intensity of peeks or even embed the whole aligned chromatogram spectra into a representative vector for similarity communication.

We thank the reviewer for this constructive suggestion. Global retention time (RT) alignment is indeed critical for computing multi-run scores, and we agree that the method used to quantify run similarity merits careful scrutiny.

In DreamDIAlignR, multi-run scores are computed by integrating single-run peak quality scores across runs. There are two components to this integration: (1) the single-run peak scores, which are derived directly from chromatographic peak features, and (2) the weights used to combine them, which are determined by global RT similarity. Therefore, our approach does not ignore chromatogram information—rather, it directly incorporates peak-level features extracted from the chromatographic signal into the scoring process.

However, we appreciate the reviewer's concern that this may not have been sufficiently clear. To clarify this point, we have revised the manuscript to explicitly explain how chromatogram-derived features contribute to both components of the multi-run score in both Results section and Methods section.

In addition, we have implemented and benchmarked three alternative global RT similarity metrics that more directly incorporate chromatographic signal characteristics: relative intensity similarity, XIC cosine similarity, and aligned XIC cosine similarity, as suggested. As shown in Supplementary Figures S10 and S11, NC similarity consistently outperformed these

alternatives on the TSTP dataset, which is why it remains the default in DreamDIAlignR. Nonetheless, these alternative metrics are now available in the software, and users can select the most appropriate method for their use cases.

3. While the weight decay parameter (k=50) effectively reduces false positives in the tested datasets, its universality across diverse experimental setups remains unclear. A sensitivity analysis or adaptive parameter selection strategy would strengthen the method's robustness.

We thank the reviewer for this insightful suggestion. In response, we have added a sensitivity analysis in Supplementary Figure S9 of the revised manuscript. We evaluated the total number of peptide identifications across a range of k values using three different datasets. In all cases, we observed a consistent trend: a sharp decrease in identifications at low k values followed by a plateau, indicating that the behavior of the parameter is generalizable across datasets.

Furthermore, when comparing identifications at k = 0 versus k = 50, we found that most of the additionally identified peptides were not new discoveries, but rather peptides that had already been identified in a few runs. This suggests that the weight decay strategy effectively downweights potentially spurious identifications with inflated multi-run scores.

To enhance robustness, we have also implemented an automated k selection strategy in DreamDIAlignR. The optimal k value is estimated by detecting the elbow point of the identification reduction curve, allowing the method to adapt to different datasets without manual tuning.

Reviewer #3

The manuscript "COMMSCHEM-25-0094-T" describes and benchmarks DreamDIAlignR, a cross-run peptide-centric tool for data-independent acquisition (DIA) analyses that integrates peptide elution behavior across runs with a deep learning peak identifier and signal alignment algorithm for consistent peak picking and FDR-controlled scoring. The integration of multi-run alignment techniques and statistical validation addresses known limitations in current DIA analyses, improving reproducibility and quantification performance. The manuscript provides an open-source implementation and publicly available datasets which enhances reproducibility and transparency. The authors systematically compare their method with established

workflows using multiple datasets. The following recommendations are provided to improve the manuscript:

1. It would be helpful for the readers to include a brief description of the pre-trained LSTM model, the first time is mentioned in the manuscript, and how it was trained.

We thank the reviewer for pointing this out. The default deep learning model used in DreamDIAlignR is the pre-trained LSTM model from our previous work, DreamDIA (Gao, M., et al., *Commun. Biol.* **4**, 1190, 2021), which was trained on approximately one million chromatographic peaks. As this model was not modified in the current study, we had initially omitted its details. To improve clarity for readers, we have now added a brief description of the model and its training procedure in the "Design of the DreamDIAlignR workflow" section.

2. Fig. 2 a-c, clarify what the gray and white boxes on the axis are representing.

We have clarified the meaning of the gray and white boxes in the figure caption, which now reads: "Gray boxes represent runs with 10% human plasma, while white boxes represent runs without human plasma."

3. Fig. 2, consider if this representation of peptide charge state is commonly used: ADGQTVSGGSILYR/3.

To improve clarity, we have revised the peptide charge state notation to the more conventional format "ADGQTVSGGSILYR^{3+}" using superscript for the charge.

4. Fig. 5 could be simplified by comparing only the results using MBR. It is clear that MBR mostly helps for each tool. Make it easier to see how your method is performing better compared to other tools. In the end, what the users want to know is which software tool provides more correct IDs and more accurate quantitation.

We thank the reviewer for this helpful suggestion. We fully agree that readers are primarily interested in understanding how DreamDIAlignR compares to other state-of-the-art tools in terms of identification accuracy and quantification performance. However, a key point of our work is that DreamDIAlignR is not simply a standalone DIA analysis tool—it is a novel MBR framework that can, in principle, be applied to any DIA software. One of its main contributions is in transforming single-run analysis into a multi-run context with the safeguard of statistical framework, significantly boosting performance.

To emphasize both its relative improvement over single-run analysis and its advantage over other software tools, we chose to present results in both contexts. That said, we appreciate the reviewer's comment and have revised the manuscript to improve clarity: we now clearly separate the internal MBR method comparison (Figure 3a and Figure 5a) from the overall performance benchmark (remaining panels in Figures 3 and 5) in distinct paragraphs. This revised structure should make it easier for readers to interpret the results and understand the specific benefits of DreamDIAlignR.

5. Fig. 6 d, the boxes inside the circles are hard to see, consider increasing the size and using a different symbol for each the tool.

In the revised manuscript, we have increased the size of the markers and used distinct symbols for each software tool to improve visibility and distinguishability in Figure 6d.

6. Software information, specify the programming language(s) of the package.

We have specified the programming language(s) used in the package in the "Software information" section of the revised manuscript.

7. Consider adding a brief discussion comparing the running times of the tools.

We have added a brief discussion of the runtime of DreamDIAlignR in the Discussion section (Figure S19).

Reviewer #1 (Remarks to the Author):

The authors have substantially improved their manuscript and I have no further comments.

Response: Thank you very much for your positive feedback and for acknowledging the improvements. We appreciate your time and effort in reviewing our work.

Reviewer #2 (Remarks to the Author):

The reviewers have addressed all my previous concerns. I would like to suggest to publish the work as is.

Response: Thank you very much for your positive feedback. We appreciate your time and effort in reviewing our work.

Reviewer #3 (Remarks to the Author):

The authors have addressed the reviewers' concerns and suggestions.

Response: Thank you very much for your positive feedback. We appreciate your time and effort in reviewing our work.
